# Double-edged sword of gonadotropin-releasing hormone (GnRH): A novel role of GnRH in the multiple beneficial functions of endometrial stem cells

**DOI:** 10.1038/s41419-018-0892-3

**Published:** 2018-08-01

**Authors:** Se-Ra Park, Ara Cho, Sung Taek Park, Chan Hum Park, Soyi Lim, Mirim Jin, Hwa-Yong Lee, In-Sun Hong

**Affiliations:** 1 Department of Health Sciences and Technology, GAIHST, Gachon University, Incheon, 21999 Republic of Korea; 2 Department of Molecular Medicine, School of Medicine, Gachon University, Incheon, 406-840 Republic of Korea; 3 Department of Obstetrics and Gynecology, Hallym University Kangnam Sacred Heart Hospital, Seoul, Korea; 4 Department of Otolaryngology-Head and Neck Surgery, Chuncheon Sacred Heart Hospital, Hallym University College of Medicine, Chuncheon, South Korea; 5 Department of Obstetrics and Gynecology, Gachon University Gil Medical Center, Incheon, Republic of Korea; 6 College of Medicine, Gachon University, Incheon, South Korea; 7 Department of Biomedical Science, Jungwon University, 85 Goesan-eup,Munmu-ro, Goesan-gun, Chungcheongbuk-do 367-700 Republic of Korea

## Abstract

Gonadotropin-releasing hormone (GnRH) stimulates the synthesis and release of gonadotropins, which induce estrogen production and subsequent ovulation. Therefore, long-term GnRH exposure to regulate ovarian hyperstimulation is recognized as the gold standard for most in vitro fertilization (IVF) strategies. However, one of the most disappointing aspects of current IVF technology is relatively low rate (between 35 and 50%) of positive pregnancy outcomes, and the major reason for this high cancellation rate has not yet been revealed. Previous studies have demonstrated that resident stem cell deficiency limits the cyclic regenerative capacity of the endometrium and subsequently increases pregnancy failure rates. Therefore, we hypothesized that long-term GnRH exposure directly damages endometrial stem cells and consequently negatively affects pregnancy outcomes in GnRH-based IVF. In addition to their well-known roles in regulating the hypothalamus-pituitary-gonadal axis, GnRH and its receptors also localize in the extra-hypothalamic endometrium, suggesting a possible non-canonical role in endometrial stem cells. Consistent with our hypothesis, we show for the first time that GnRH suppresses the multiple beneficial functions of endometrial stem cells via the PI3K/Akt signaling pathway in vitro and in vivo. To the best of our knowledge, this is the first study to focus on the direct effects of GnRH on the regenerative potential of stem cells, and the findings will facilitate the development of more promising IVF strategies.

## Introduction

GnRH is the central neuroendocrine regulator of reproductive function in vertebrates^1,2^. This decapeptide is secreted by neurons within the hypothalamus and delivered to the anterior pituitary. GnRH acts on the pituitary to stimulate the synthesis and release of gonadotropins [luteinizing hormone (LH) and follicle-stimulating hormone (FSH)], which enable the recovery of a larger number of oocytes^3^. Therefore, long-term exogenous GnRH exposure to stimulate the ovary is recognized as the gold standard for most in vitro fertilization (IVF) strategies^4^. However, the implantation and clinical pregnancy rates in infertile patients undergoing the GnRH agonist protocol are only 5 and 15%, respectively^5^. Unfortunately, the major reason for these high cancellation rates with GnRH-based IVF therapy has not yet been revealed.

Successful implantation and subsequent pregnancy largely depend on reciprocal interactions between the embryo and endometrium (innermost lining of the uterus)^6^. The human endometrium is an extraordinarily dynamic tissue that grows ~7 mm within 1 week and develops a rich blood supply for potential embryo implantation in every menstrual cycle^7^. Endometrial regeneration repeats for ~500 cycles of growth and shedding in a tightly controlled manner during a woman’s reproductive life^8^. Additionally, the physiological features or responses of endometrial cells to exogenous stimuli vary depending on the phase of menstrual cycle as well as the status of menopause. For example, the gene expression patterns of key proteins regulating embryo implantation vary through the menstrual cycle^9^. Menopausal status also strongly influences the levels of steroid action regulators with subsequent morphological endometrial alterations^10^. Like many other human tissues, resident stem cells are responsible for this cyclic regeneration of endometrial function and tissue repair^11,12^. Moreover, implantation requires the constant activation and recruitment of local stem cells that can differentiate into specialized endometrial cell types prior to and during pregnancy^13^. Interestingly, recent work revealed that stem cell deficiency limits the cyclic regenerative capacity of the endometrium and subsequently increases pregnancy failure rates^13^.

Previous studies have shown that in addition to their well-known roles in regulating the hypothalamus-pituitary-gonadal axis, GnRH and its receptors also localize in extra-hypothalamic reproductive tissues, such as the placenta^14^, ovary^15^, and endometrium^16^. More importantly, the low implantation and clinical pregnancy rates with GnRH-based IVF protocols could be associated with various side effects of long-term GnRH exposure. Indeed, Weng et al. raised concerns regarding unfavorable effects of GnRH exposure on endometrial epithelial cells^17^. Consistent with these results, Ersoy et al. revealed that long-term treatment of GnRH analog (leuprolide acetate) significantly reduced the recruitment and growth of bone marrow–derived stem cells (BMDSCs) engraftment in vivo^18^. However, it is unclear whether these reduced stem cell engraftment is due to the direct inhibitory effect of GnRH or the indirect effect of GnRH-induced suppression of estrogen in mice. In this context, we therefore hypothesized in present study that exogenous GnRH exposure directly damages endometrial stem cells and consequently reduces favorable pregnancy outcomes with GnRH-based IVF treatment. However, the direct effects of GnRH on endometrial stem cells and the underlying mechanisms involved remain unknown.

Consistent with our hypothesis, we show for the first time that the GnRH receptor (GnRH-R) is more highly expressed in endometrial stem cells than in terminally differentiated fibroblasts and that GnRH acts as a potent inhibitory factor for multiple endometrial stem cell functions, such as proliferation differentiation, and migration in vitro and in vivo. We subsequently explored the molecular mechanism underlying these inhibitory effects of GnRH on various endometrial stem cell functions. Strikingly, GnRH suppresses survival pathways, such as the PI3K/Akt signaling cascade, that are involved in various physiological functions, including stem cell proliferation^19,20^, differentiation^19,21,22^, and migration^19,23^. Taken together, these findings suggest that in addition to its previously reported canonical hypothalamic function, GnRH acts as an exogenous damaging factor to directly suppress the regenerative capacity of the endometrium by inhibiting multiple beneficial functions of endometrial stem cells via the PI3K/Akt signaling pathway. Increased understanding of this molecular cascade may facilitate the development of promising therapeutic strategies that effectively improve the success rate of current fertility treatments.

## Results

### GnRH directly inhibits multiple beneficial functions of endometrial stem cells in vitro

**Fig. 1:**
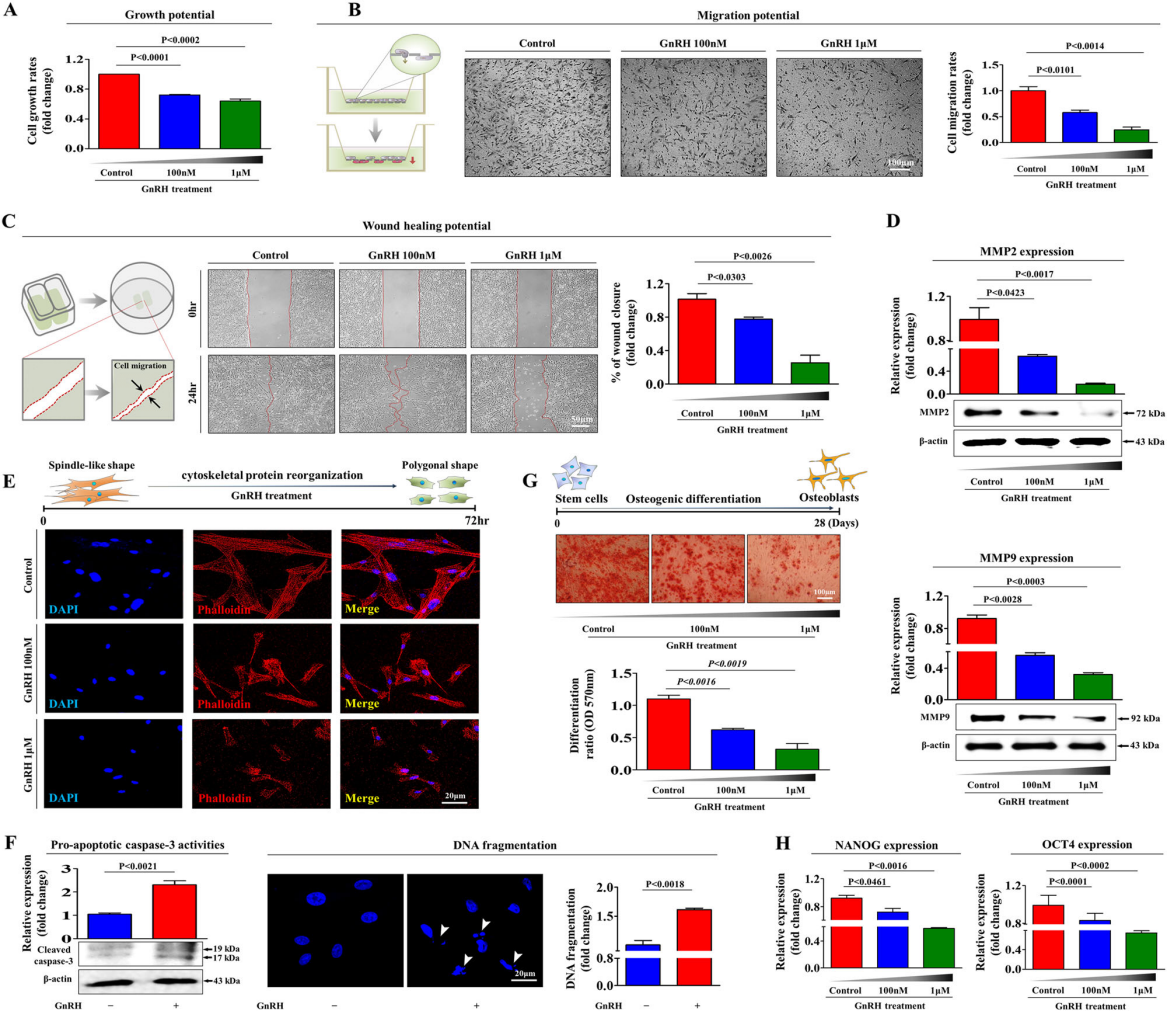
GnRH suppresses multiple beneficial functions of endometrial stem cells in vitro. The inhibition of endometrial stem cell viability by 1 µM GnRH at 72 h was determined by an MTT assay. Stem cell viability (%) was calculated as a percent of the vehicle control (**a**). Endometrial stem cells were treated with GnRH for 72 h, and the effect of GnRH on stem cell migration ability was then evaluated using the transwell migration assay. GnRH treatment significantly decreased stem cell migration across the membrane compared with the negative control (**b**). The effects of GnRH on stem cell migration were further evaluated using a scratch assay. The migration of stem cells treated with GnRH was slower than that of cells treated with vehicle. (**c**). The relative expression levels of key positive regulators of cell migration (MMP-2/9) were assessed using western blotting (**d**). GnRH-induced actin filament disorganization and morphological changes in stem cells were visualized by staining actin filaments with phalloidin (**e**). Elevated levels of cleaved caspase-3 following GnRH treatment were assessed by western blotting. GnRH-induced apoptotic DNA fragmentation and condensation were visualized using DAPI staining (**f**). Confluent stem cells were cultured in osteogenic medium with or without GnRH. The effects of GnRH on osteoblast differentiation were determined by alizarin red staining. The relative quantification of calcium mineral content was performed by measuring the absorbance at 570 nm (**g**). Real-time PCR results showed the changes in the expression of the stem cell markers NANOG and OCT4 after GnRH treatment for 72 h (**h**). DAPI staining was used to label the nuclei. β-actin was used as the internal control. The data are presented as the mean ± SD of three independent experiments

We first isolated endometrial stem cells from human endometrial tissue (Supplementary Figure 1a) and then characterized their biological properties by using multiple negative and positive stem cell surface markers such as CD34, CD44, CD45, CD73, CD105, CD140B, CD146, and W5C5 (Supplementary Figure 1B). The capacity of endometrial stem cells to differentiate into multiple lineages was evaluated by inducing adipogenic and osteogenic differentiation (Supplementary Figure 1C). As GnRH might play a role as an exogenous factor that damages the endometrium, we investigated whether GnRH inhibits the various beneficial functions of endometrial stem cells in vitro. We first examined the proliferative response of stem cells to GnRH and found steadily decreasing growth rates in endometrial stem cells treated with GnRH compared with non-treated control cells (Fig. 1a). More strikingly, transwell migration (Fig. 1b) and wound healing (Fig. 1c) assays revealed the inhibitory effect of GnRH on the migratory ability of endometrial stem cells in vitro. To further confirm the inhibitory effect of GnRH on endometrial stem cell migration, western blotting was used to evaluate the expression levels of matrix metalloproteinase 2 (MMP-2) and MMP-9, which play important roles in regulating cell migration (Fig. 1d). Previous studies have indicated that the actin cytoskeleton is involved in cell migration via pushing or pulling the substrate near the plasma membrane^24^. Consistently, phalloidin staining of actin filaments revealed a strong correlation between GnRH treatment and greater actin cytoskeleton disorganization (Fig. 1e), suggesting that the markedly decreased migratory capability of GnRH-treated stem cells may be related to actin cytoskeleton disorganization. GnRH also increased pro-apoptotic caspase 3 activity and subsequent DNA fragmentation (Fig. 1f). Importantly, GnRH significantly decreased multi-lineage differentiation potential toward osteoblasts in vitro (Fig. 1g). Consistently, the expression levels of the pluripotency-associated factors NANOG and OCT4 were significantly decreased by GnRH treatment (Fig. 1h). FSH is routinely administered during in vitro fertilization treatment to stimulate the development of multiple follicles after pituitary downregulation with GnRH agonist^25^. Therefore, to further determine whether our in vitro findings can be applied to the clinical setting, we additionally investigated whether FSH affects GnRH-mediated effects on the multiple functions of endometrial stem cells. Interestingly, the GnRH-induced suppression of stem cell proliferation (Supplementary Figure 2A), apoptosis (Supplementary Figure 2B and C), the expression levels of the pluripotency-associated factors (Supplementary Figure 2D), and migration (Supplementary Figure 2E) was barely affected by FSH co-treatment in vitro. Taken together, these results suggest that GnRH suppresses multiple beneficial functions, such as proliferative, migratory, and multi-lineage differentiation potential, of endometrial stem cells in vitro. To confirm whether this effect is specific to endometrial stem cells or occurs in all stem cells, we isolated stem cells from human adipose tissues and treated them with various doses of GnRH. We isolated stem cells from human adipose tissue using our standard method (Supplementary Figure 3A) and then characterized the biological properties of these stem cells using a combination of various positive and negative stem cell surface markers (Supplementary Figure 3B). The ability of adipose-derived stem cells to differentiate into various tissue lineages was confirmed by inducing osteoblast and adipocyte differentiation in vitro (Supplementary Figure 3C). Consistent with the results with endometrial stem cells, those with human adipose tissue-derived stem cells showed that GnRH acts as a potent inhibitory factor of multiple beneficial functions, such as proliferation, differentiation, and migration (Supplementary Figure 4A–E).

### GnRH inhibits multiple endometrial stem cell functions through its functional receptor

**Fig. 2:**
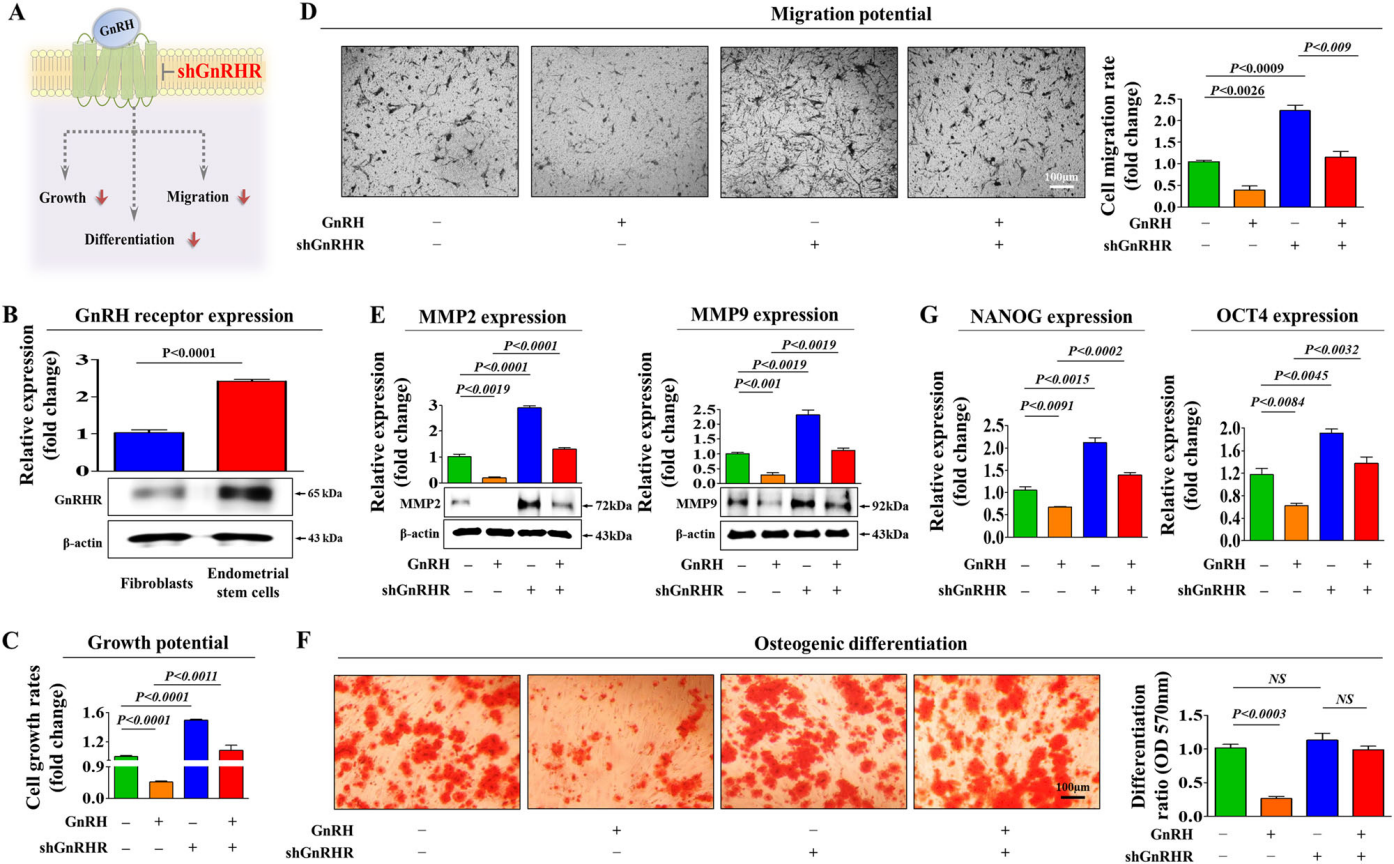
GnRH inhibits multiple beneficial stem cell functions through its cognate receptor. Schematic representation of the experimental protocol as described in the materials and methods section (a). Fibroblasts and endometrial stem cells were incubated under standard culture conditions, and the expression levels of GnRH-R were assessed by western blotting (**b**). Endometrial stem cells were treated with 1 µM GnRH alone or in combination with shRNA targeting GnRH-R; subsequent changes in cell viability were measured by an MTT assay (**c**). Changes in migratory capacity were measured by the transwell assay (**d**) and western blotting for MMP-2 and MMP-9 **(e)**. The ability of GnRH-R knockdown to attenuate the GnRH-induced suppression of osteoblast differentiation was determined by alizarin red staining. The relative quantification of calcium mineral content was performed by measuring the absorbance at 570 nm (**f**). Real-time PCR results showed the changes in the expression of the stem cell markers NANOG and OCT4 after GnRH treatment for 72 h alone or in combination with GnRH-R shRNA (**g**). β-actin was used as the internal control. The data are presented as the mean ± SD of three independent experiments

The effects of GnRH are mediated by a cell surface receptor (GnRH-R) belonging to the G protein-coupled receptor superfamily^26,27^. Therefore, we first assessed whether isolated human endometrial stem cells express GnRH-R and whether the expressed receptors are functional. We confirmed that endometrial stem cells indeed expressed GnRH-R, and interestingly, GnRH-R expression was higher in stem cells than in terminally differentiated fibroblasts (Fig. 2a, b). We additionally analyzed whether both human and mouse adipose-derived stem cells express GnRH receptor. Importantly, both stem cells expressed GnRH receptors although the expression was more marked in human stem cells than mouse stem cells (Supplementary Figure 5). Next, to further investigate whether GnRH-R acts as a functional receptor for GnRH in endometrial stem cells, stable GnRH-R was stably knocked down by transfecting cells with shRNA targeting GnRH-R (Supplementary Figure 6A–C). Importantly, the GnRH-induced suppression of stem cell proliferation (Fig. 2c) and migration (Fig. 2d, e) was significantly attenuated by GnRH-R depletion. In addition, the GnRH-induced effects on osteogenic differentiation were markedly disrupted by GnRH-R knockdown (Fig. 2f). Consistently, the inhibitory effects of GnRH on pluripotency-associated factors (NANOG and OCT4) were also significantly attenuated by GnRH-R depletion (Fig. 2g). Taken together, these results suggest that GnRH-R may serve as a functional receptor to regulate the multiple inhibitory effects of GnRH on endometrial stem cell functions.

### PI3K/Akt signaling mediates the GnRH-induced inhibitory effects on endometrial stem cells

**Fig. 3:**
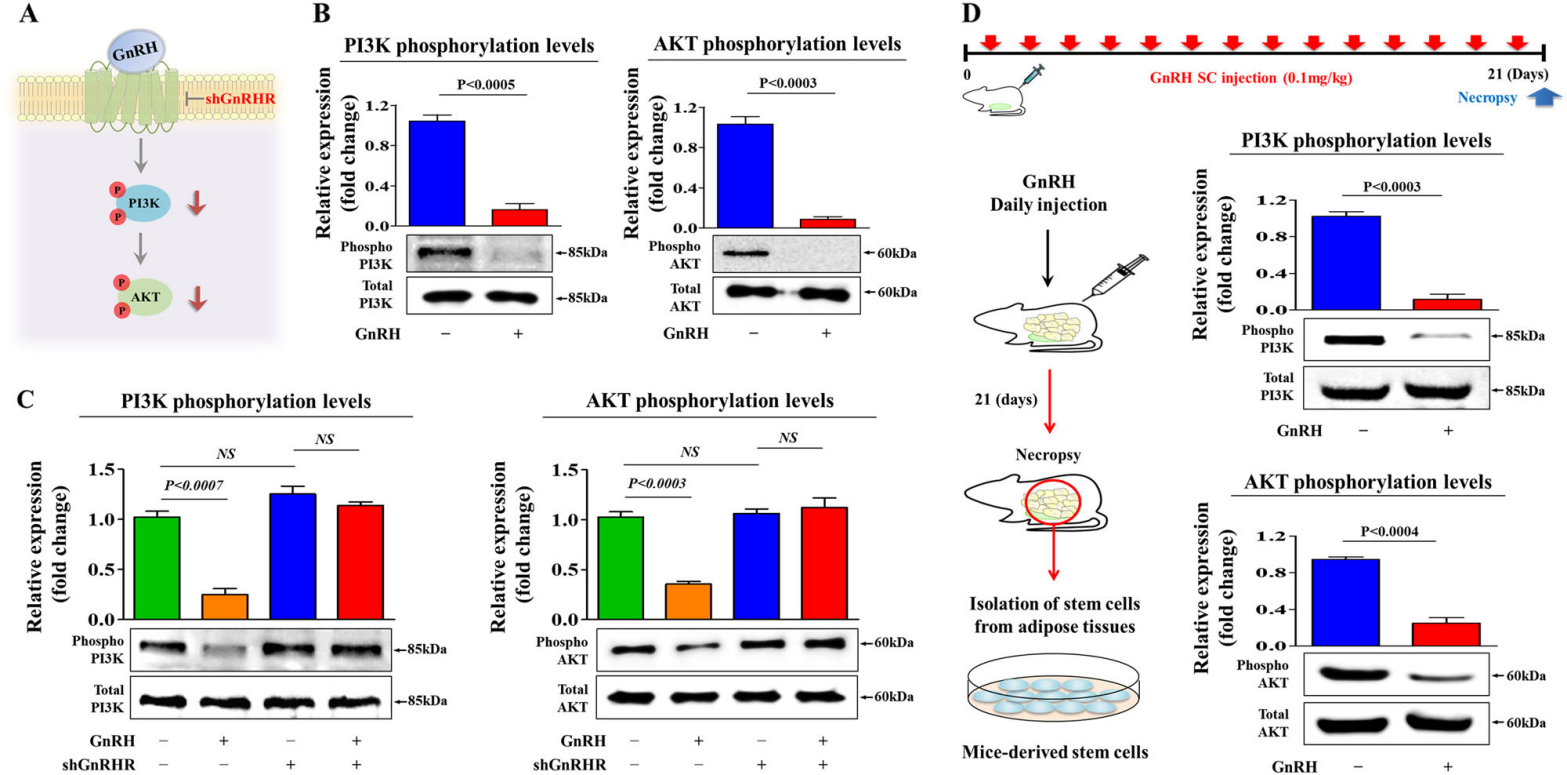
The inhibitory effects of GnRH on PI3K/Akt signaling in vitro and in vivo. Schematic representation of the experimental protocol as described in the materials and methods section (**a**). Endometrial stem cells were treated for 10 min with or without GnRH (1 µM). The cells were then lysed, and the protein contents were analyzed by western blotting using antibodies targeting the phosphorylated forms of PI3K and Akt. The phosphorylation levels of these signaling molecules were significantly decreased in stem cells treated with GnRH (**b**). Endometrial stem cells were treated with 1 µM GnRH alone or in combination with shRNA targeting GnRH-R; subsequent changes in the phosphorylation levels of PI3K and Akt were measured via western blotting (**c**). Mice were treated daily for 21 days with GnRH (0.1 mg/kg, subcutaneously) or vehicle (PBS). Stem cells were isolated from mouse adipose tissues, and changes in the phosphorylation levels of PI3K and Akt were measured via western blotting (**d**). β-actin was used as the internal control. The data are presented as the mean ± SD of three independent experiments

**Fig. 4:**
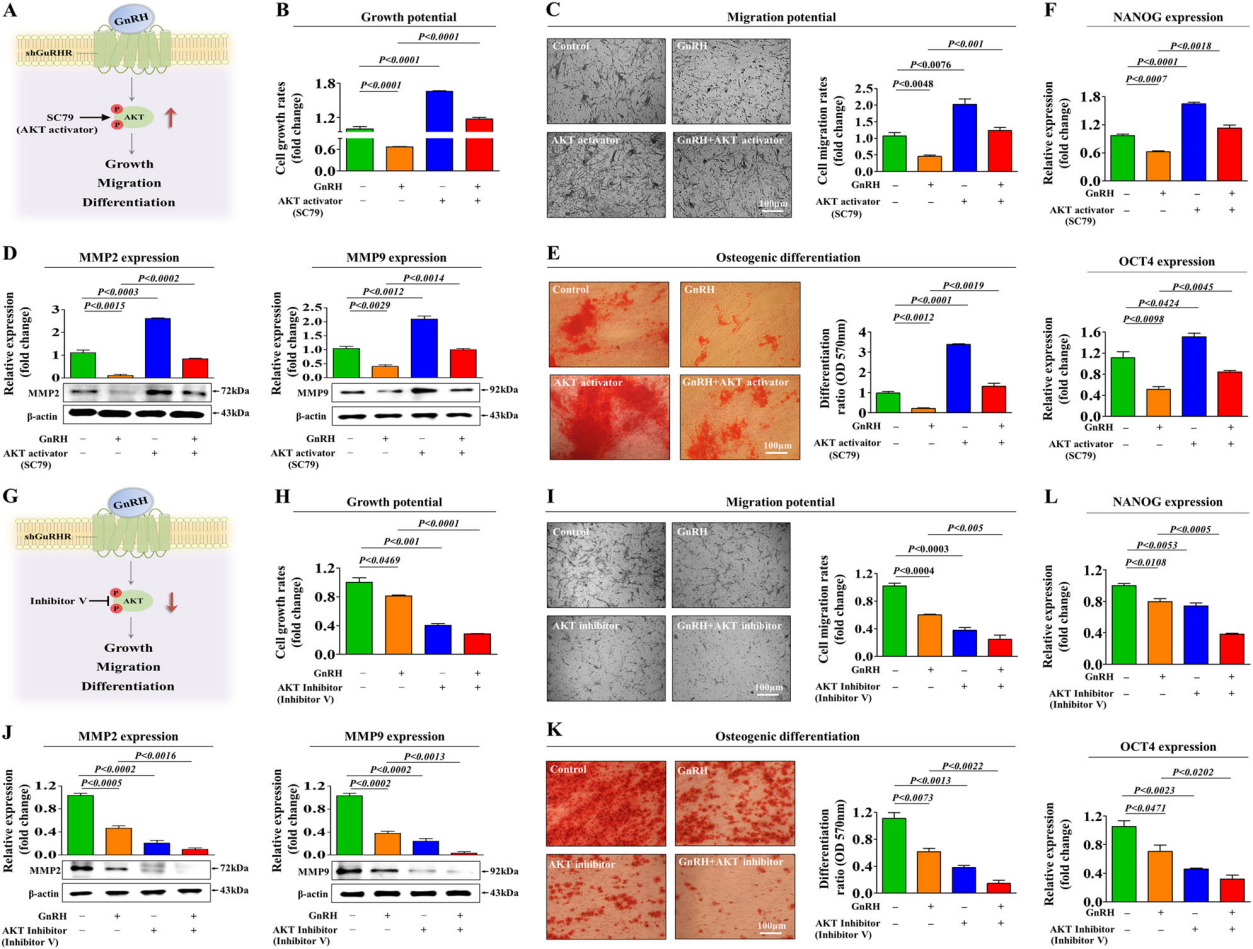
Activation or inhibition of Akt signaling attenuates the GnRH-induced effects on multiple stem cell functions. Schematic representation of the experimental protocol as described in the materials and methods section (**a**). Endometrial stem cells were pretreated with the Akt activator SC79 (10 µM) for 24 h prior to treatment with 1 µM GnRH for 48 h, and the changes in cell viability were determined by an MTT assay. Stem cell viability (%) was calculated as a percent of the vehicle control (**b**). The changes in migratory capacity were measured via the transwell assay **(c)** and western blotting for MMP-2 and MMP-9 (**d**). The changes in osteoblast differentiation were determined by alizarin red staining. The relative quantification of calcium mineral content was performed by measuring the absorbance at 570 nm (**e**). The ability of SC79 to attenuate the GnRH-induced inhibition of stem cell marker expression (NANOG and OCT4) was determined by real-time PCR (**f**). Schematic representation of the experimental protocol as described in the materials and methods section (**g**). Endometrial stem cells were pretreated with Akt inhibitor V (10 µM) for 24 h prior to treatment with 1 µM GnRH for 48 h, and the changes in cell viability were determined by an MTT assay. Stem cell viability (%) was calculated as a percent of the vehicle control (**h**). The changes in migratory capacity were measured via the transwell assay (**i**) and western blotting for MMP-2 and MMP-9 (**j**). The changes in osteoblast differentiation were determined by alizarin red staining. The relative quantification of calcium mineral content was performed by measuring the absorbance at 570 nm (**k**). The synergism between Akt inhibitor V and GnRH in inhibiting stem cell marker expression (NANOG and OCT4) was determined by real-time PCR (**l**). β-actin was used as the internal control. The data are presented as the mean ± SD of three independent experiments

To investigate the underlying molecular mechanisms of the GnRH-induced inhibitory effects, we examined the effect of GnRH on PI3K/Akt signaling, which has been associated with stem cell maintenance^28^, differentiation^29^, and migration^30^. Using western blot analysis, we evaluated whether GnRH treatment was sufficient to activate the PI3K/Akt signaling cascade in endometrial stem cells. Importantly, the phosphorylation levels of signaling molecules within this pathway were significantly decreased in GnRH-treated cells (Fig. 3a, b). We then examined the effect of GnRH-R knockdown on GnRH-induced PI3K/Akt signaling. As expected, the GnRH-induced suppression of PI3K/Akt signaling was significantly attenuated by GnRH-R depletion (Fig. 3c). Then, to determine whether GnRH successfully inhibits these signaling pathways in vivo, we investigated the phosphorylation levels of these signaling components in animal models with or without GnRH treatment. Consistent with the in vitro results, the in vivo results revealed that GnRH treatment significantly decreased PI3K/Akt signaling in mouse-derived stem cells (Fig. 3d). Next, to determine whether activation of this signaling pathway attenuates the GnRH-induced inhibitory effects on multiple beneficial functions of endometrial stem cells, we evaluated the effects of the Akt activator SC79 (Fig. 4a) or Akt inhibitor V (Fig. 4g) on endometrial stem cell growth, migration and differentiation with or without GnRH treatment. Indeed, the GnRH-induced suppression of growth (Fig. 4b), migration (Fig. 4c, d), differentiation potential (Fig. 4e), and pluripotency-associated factors (Fig. 4f) was markedly attenuated by treatment with SC79. Consistently, Akt inhibitor V synergized with GnRH in inhibiting the growth (Fig. 4h), migration (Fig. 4I, j), differentiation potential (Fig. 4k), and pluripotency-associated factors (Fig. 4l) of endometrial stem cells. These results suggest that the PI3K/Akt signaling cascade may be involved in the GnRH-induced inhibitory effects on multiple beneficial functions of endometrial stem cells.

### Proteome profiler analysis of GnRH-induced expression of secreted proteins and interconnected signaling networks

**Fig. 5:**
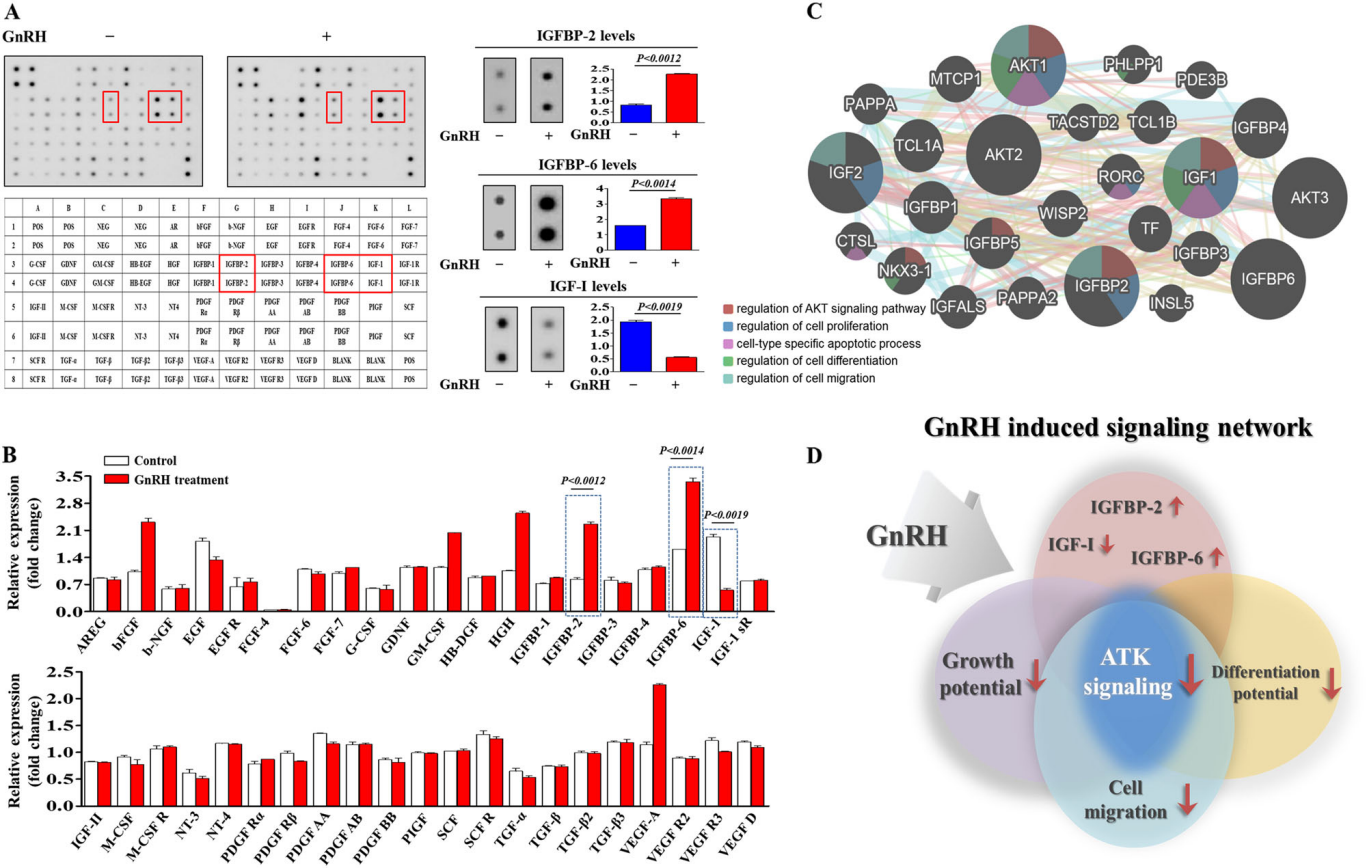
GnRH-induced secreted proteins are associated with PI3K/Akt signaling. Human growth factor antibody array analysis was performed using GnRH-treated and control samples. The membrane was printed with antibodies for 40 growth factors, cytokines, and receptors, with four positive and four negative controls in the upper and lower left corners. Three growth factors or related proteins (IGF-I, IGFBP-2, and IGFBP-6) were markedly enriched in the GnRH-treated groups compared with the control groups (**a**, **b**). Signaling network analysis was performed using GeneMANIA (http://www.genemania.org) to predict the connections between the three growth factors and PI3K/Akt signaling. The results revealed a positive relationship between each of the three factors (IGF-I, IGFBP-2, and IGFBP-6) and PI3K/Akt signaling (**c**, **d**). The data are presented as the mean ± SD of three independent experiments

To identify major secreted factors responsible for the inhibitory effects of GnRH, we analyzed the GnRH-induced expression of secreted proteins using antibody arrays for multiple common cytokines/growth factors. In duplicate experiments, we detected 40 proteins in both GnRH-treated endometrial stem cells and non-treated control cells. The expression levels of two growth factors, namely, insulin-like growth factor-binding protein 2 and 6 (IGFBP-2 and 6), major suppressors of IGF-I-induced Akt signaling^31,32^, were elevated substantially by GnRH treatment, whereas the levels of other factors showed only minor enhancements (Fig. 5a, b). Consistently, the expression of insulin-like growth factor-I (IGF-I), a major activator of PI3K/Akt signaling, was decreased significantly by GnRH (Fig. 5a, b). These results suggest that these factors may be at least partly responsible for the GnRH-induced suppression of PI3K/Akt signaling and the subsequent inhibitory effects on beneficial multiple functions of endometrial stem cells. Therefore, we analyzed the activation state of PI3K/Akt signaling and the expression levels of IGFBP-2, IGFBP-6, and IGF-I using GeneMANIA (http://www.genemania.org) to evaluate interconnected signaling networks governing proliferation, migration, and differentiation potential (Fig. 5c). To find gene interactions, we considered several factors, including co-expression, co-localization, and genetic interactions. The results revealed a strong relationship between the activation state of PI3K/Akt signaling and the expression levels of the three most prominent factors, IGFBP-2, IGFBP-6, and IGF-I (Fig. 5d).

### GnRH inhibits multiple beneficial functions and the regenerative potential of stem cells in vivo

**Fig. 6:**
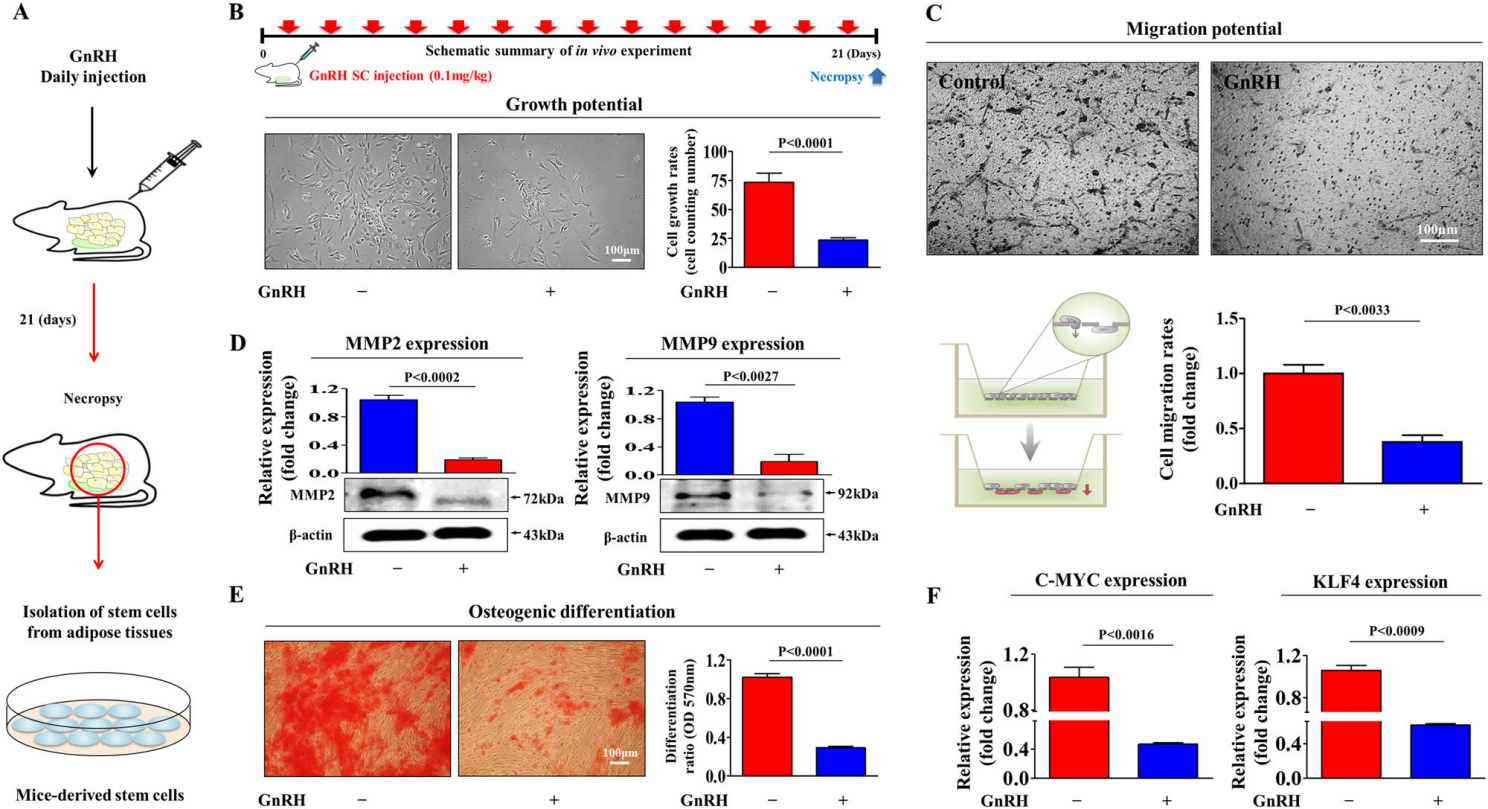
GnRH inhibits multiple beneficial functions of stem cells in vivo. Schematic representation of the experimental protocol as described in the materials and methods section (**a**). Mice were treated daily for 21 days with GnRH (0.1 mg/kg, intraperitoneally) or vehicle (PBS). Stem cells were isolated from mouse adipose tissue, and changes in cell viability were determined by an MTT assay. Stem cell viability (%) was calculated as a percent of the vehicle control (**b**). The changes in migratory capacity were measured via the transwell assay (**c**) and western blotting for MMP-2 and MMP-9 (**d**). The changes in osteoblast differentiation were determined by alizarin red staining. The relative quantification of calcium mineral content was performed by measuring the absorbance at 570 nm (**e**). Real-time PCR results showed the changes in the expression of the mouse stem cell markers C-MYC and KLF4 after GnRH treatment in vivo (**f**). β-actin was used as the internal control. The data are presented as the mean ± SD of three independent experiments

Our in vitro data suggested that long-term exposure to exogenous GnRH to stimulate the ovary may suppress the regenerative potential of stem cells in vivo during IVF therapy. Therefore, we next investigated whether GnRH affects multiple beneficial functions of endometrial stem cells and their regenerative potential in an animal model. To exactly mimic clinical conditions, mice received an intraperitoneal injection of GnRH (0.1 mg/kg) on 21 consecutive days, similar to the dosing schedule in GnRH-based IVF protocols, and stem cells were then isolated from adipose tissue (Fig. 6a). Consistent with the in vitro results, the in vivo results revealed that GnRH significantly decreased the growth potential of stem cells (Fig. 6b). Strikingly, the transwell migration assay showed the inhibitory effect of GnRH on the migratory ability of endometrial stem cells in vivo (Fig. 6c). To further confirm the stimulatory effect of GnRH on stem cell migration, western blotting was used to evaluate MMP-2/9 expression levels (Fig. 6d). Importantly, GnRH significantly decreased multi-lineage differentiation potential toward osteoblasts in vivo (Fig. 6e). Consistently, the expression levels of the pluripotency-associated factors C-MYC and KLF4 were significantly decreased by GnRH (Fig. 6f). Furthermore, we conducted the experiments to evaluate whether GnRH-induced damaging effect on endometrial stem cells could affect the histological conditions of uterine endometrium in vivo. Importantly, histological examination revealed that intraperitoneal injection of GnRH (0.1 mg/kg) on 21 consecutive days clearly narrowed the endometrial functional layer with degenerative changes and a loss of endometrial glands (Supplementary Fig. 7). The results revealed a strong relationship between the GnRH-induced damaging effect on endometrial stem cells and degenerative changes of uterine endometrium in vivo. Furthermore, we intraperitoneally injected GnRH (0.1 mg/kg) with FSH (mg/kg) into mice on 21 consecutive days and stem cells were then isolated from endometrial tissues. Interestingly, the GnRH-induced suppression of stem cell proliferation (Supplementary Fig. 8A), apoptosis (Supplementary Fig. 8B and C), the expression levels of the pluripotency-associated factors (Supplementary Fig. 8D), and migration (Supplementary Fig. 8E) was barely affected by FSH co-treatment in vivo.

## Discussion

One of the most disappointing aspects of current assisted reproductive technology is the significantly low implantation and pregnancy success rates, which are only 5.26% and 15.82%, respectively^5^. Patients suffering from recurrent implantation failure or repeated miscarriage must undergo an intensive investigation of endometrial receptivity, which is presumed to be a key step in the embryo implantation process^33,34^. The human endometrium undergoes extensive stromal proliferation and angiogenesis in order to facilitate successful implantation, and the entire endometrial surface of the uterine cavity rapidly regenerates following each menstrual cycle^35^. This remarkable regenerative ability of the endometrium is absolutely necessary for successful reproduction. Endometrial stem cells likely residing in the basalis (basal layer), which may not be shed during each menstrual cycle, are responsible for the remarkable regenerative capacity of the endometrium^36^. Lucas et al. revealed that recurrent pregnancy loss is strongly associated with a relative lack of endometrial stem cells^13^. Importantly, no highly clonogenic endometrial stem cells were identified in 42% of recurrent miscarriage endometrial biopsies compared with 11% of normal biopsies^13^. Because of the close association between endometrial stem cells and endometrium reconstruction, increased endometrial stem cell damage may decrease implantation success and result in a lower pregnancy rate. Interestingly, the cellular senescence of endometrial stem cells triggers a chronic inflammatory response, which is associated with susceptibility to recurrent miscarriages^37^. Therefore, intensive study of endometrial stem cells may provide new insights into the leading causes of pregnancy failure. At this stage, new challenging questions have arisen regarding the potential mechanisms underlying the relatively low success rate of GnRH-based assisted reproductive technology.

During controlled ovarian hyperstimulation (COH) in most IVF strategies, GnRH stimulates the synthesis and release of gonadotropins, LH and FSH, which induce estrogen production and subsequent ovulation. Therefore, the major benefits of a GnRH-based protocol seem to be the larger number of oocytes recovered and the lower cycle cancellation rate, leading to improved pregnancy rates. In addition to their well-known roles in regulating the hypothalamus-pituitary-gonadal axis, GnRH and its receptors are also expressed in extra-hypothalamic reproductive tissues, such as the endometrium^38^, ovaries^39^, oviducts^40^, placenta^41^, prostrate^42^, and testes^43^, suggesting a possible non-canonical role in reproductive tissues. Indeed, a series of studies have shown that GnRH acts as a negative regulator of breast^44^, endometrial^45^, ovarian^46^ and prostate^47^ cancer cell growth. Additionally, previous studies have demonstrated that GnRH induces apoptosis and reduces the proliferation of both eutopic and ectopic endometrial cells^17,48,49^. Therefore, we hypothesized that long-term GnRH exposure directly damages endometrial stem cells and consequently reduces positive pregnancy outcomes of GnRH-based IVF protocols. However, the direct effects of GnRH on endometrial stem cells and the underlying mechanisms remain unknown. Consistent with our hypothesis, GnRH suppressed multiple beneficial functions, such as the proliferative, migratory, and multi-lineage differentiation potential, of endometrial stem cells in vitro (Fig. 1a–h) and in vivo (Fig. 6a–f). More importantly, GnRH significantly suppressed various functions of human adipose tissue-derived stem cells (Supplementary Figure 4a–e), suggesting that GnRH may act as a universal inhibitory factor for stem cells in multiple tissue types. Although FSH is routinely administered during in vitro fertilization treatment to stimulate the development of multiple follicles after pituitary downregulation with GnRH agonist^25^, treating cells with FSH after GnRH is different from clinical situation in vivo to produce high level estrogen which can promote the stem cells proliferation and differentiation. Therefore, while we have confirmed that GnRH-induced suppression of various stem cell functions was barely affected by FSH co-treatment (Supplementary Figure 2A-E), whether estrogen affects GnRH-mediated effects on the functions of endometrial stem cells in vivo remains unknown and this warrants further investigation in future studies. Inconsistent with our study, van der Linden revealed that the addition of GnRH agonist to progesterone improved live birth or ongoing pregnancy rates of IVF^50^. Therefore, while we have confirmed that GnRH-induced suppression of endometrial stem cell functions under certain conditions, whether GnRH exerts same effects on the stem cells in different IVF conditions remains unknown and this warrants further investigation in future studies.

A cellular receptor through which GnRH exerts its biological functions in stem cells has not previously been identified. Here, we found that endometrial stem cells indeed expressed GnRH-R, which belongs to the G protein-coupled receptor superfamily; interestingly, GnRH-R expression was higher in stem cells than in terminally differentiated fibroblasts, suggesting that stem cells are likely to be more sensitive to GnRH than are differentiated cells. Importantly, the GnRH-induced suppressive effects on stem cell proliferation (Fig. 2c), migration (Fig. 2d, e), and differentiation potential (Fig. 2f and G) were significantly attenuated by GnRH-R depletion. These results suggest that GnRH-R may serve as a functional receptor to regulate the multiple inhibitory effects of GnRH on endometrial stem cell functions. Generally, the PI3K/Akt signaling pathway is preferentially activated by mitogens and growth factors in multiple stem cell types^51,52^. We therefore investigated whether GnRH exerts its stem cell suppressive function via inhibition of the PI3K/Akt signaling pathway. Indeed, GnRH-induced suppression of PI3K/Akt signaling was significantly attenuated by GnRH-R depletion (Fig. 3c). Furthermore, the GnRH-induced suppression of stem cell growth (Fig. 4b), migration (Fig. 4c, d), differentiation potential (Fig. 4e), and pluripotency-associated factors (Fig. 4f) was greatly attenuated by treatment with the Akt activator SC79. These results suggest that the PI3K/Akt signaling cascade may be involved in the GnRH-induced inhibitory effects on multiple beneficial functions of endometrial stem cells.

Taken together, these findings suggest that in addition to its previously reported canonical activities in the hypothalamic-pituitary-gonadal axis, GnRH has novel biological activity in extra-hypothalamic reproductive tissues. To the best of our knowledge, this is the first study to focus on the direct effects of GnRH on the regenerative potential of stem cells and to provide evidence that GnRH suppresses multiple beneficial functions of endometrial stem cells via the PI3K/Akt signaling pathway. This knowledge could contribute to a better understanding of the mechanisms underlying the currently poor pregnancy outcomes with GnRH-based IVF. Furthermore, our findings may facilitate the development of more promising IVF strategies with an improved success rate.

## Materials and Methods

### Isolation and culture of human endometrial stem cells

Human endometrial stem cells were obtained from endometrial tissues of uterine fibroid patients with written informed consent from the patients and approval of the Hallym University Kangnam Sacred Heart Hospital Institutional Review Board (IRB No: HKS 2017-03-001). All of the human-related experiments were approved and conducted in accordance with the Gachon University Institutional Review Board (IRB No: GAIRB2015-104). Endometrial tissue was minced into small pieces, and then the small pieces were digested in DMEM containing 10% FBS and 250 U/ml type I collagenase for 5 h at 37 °C in a rotating shaker. The digestion mixture was then filtered through a 40 µm cell strainer to separate stromal like stem cells from epithelial gland fragments and undigested tissue. Isolated cells were then cultured in EBM-2 medium (Lonza) with EGM-2 supplements at 37 °C and 5% CO_2_.

### Cell proliferation assay

The MTT assay was used to determine the anti-proliferative capacity of GnRH, according to the manufacturer’s protocol. Cells (1 × 10^4^ cells per well) were seeded in 96-well plates. After 24 h of incubation, the cells were treated with GRS or vehicle for 72 h. The viable cells were measured at 570 nm using a VersaMax microplate reader.

### In vitro cell migration assay

Cells were plated at 1 × 10^5^ cells/well in 200 μL of culture medium in the upper chambers of transwell permeable supports (Corning Inc., Corning, NY, USA) to track the migration of cells. The transwell chambers had 8.0 μm pores in 6.5-mm diameter polycarbonate membranes and used a 24-well plate format. Non-invading cells on the upper surface of each membrane were removed by scrubbing with laboratory paper. Migrated cells on the lower surface of each membrane were fixed with 4% paraformaldehyde for 5 min and stained with hematoxylin for 15 min. Later, the number of migrated cells was counted in three randomly selected fields of the wells under a light microscope at 50X magnification. To calculate the chemotactic index, the number of cells that migrated in response to the treatment of GnRH was divided by the number of spontaneously migrating cells.

### Scratch test assay

Tissue culture dishes (60 mm in diameter) were seeded with 1.0 × 10^5^ cells and maintained until the cell monolayers were confluent. These confluent monolayers were then scratched with a sterile pipette tip to generate a 12-mm-wide area without cells. The cell surface was then washed 3 times with PBS to remove dislodged cells. The cells were then incubated at 37 °C and 5% CO_2_ with GnRH or vehicle for 24 h. Cells then were fixed with 4% paraformaldehyde for 15 min and washed twice with PBS. Wound closure was monitored by collecting digitized images at 0 and 24 h after the scratch was made.

### Protein isolation and western blot analysis

The protein expression levels were determined by western blot analysis as previously described^53^. Cells were lysed in a buffer containing 50 mM Tris, 5 mM EDTA, 150 mM NaCl, 1 mM DTT, 0.01% NP 40, and 0.2 mM PMSF. The protein concentrations of the total cell lysates were measured by using bovine serum albumin as a standard. Samples containing equal amounts of protein were separated via sodium dodecyl sulfate-polyacrylamide gel electrophoresis (SDS-PAGE) and then transferred onto nitrocellulose membranes (Bio-Rad Laboratories). The membranes were blocked with 5% skim milk in Tris-buffered saline containing Tween-20 at RT. Then, the membranes were incubated with primary antibodies against β-actin (Abcam, MA, USA, ab189073), MMP-2 (Cell signaling #4022), MMP-9 (Cell Signaling #13667), total PI3K (Cell Signaling #4292), phospho-PI3K (Cell Signaling #4228), total Akt (Cell Signaling #4491), phospho-Akt (Cell Signaling #4060) overnight at 4 °C and then with HRP-conjugated goat anti-rabbit IgG (BD Pharmingen, San Diego, CA, USA, 554021) and goat anti-mouse IgG (BD Pharmingen, 554002) secondary antibodies for 60 min at RT. Antibody-bound proteins were detected using ECL reagents.

### Immunofluorescent staining

Samples were fixed with 4% paraformaldehyde for fluorescent staining. Samples were permeabilized with 0.4 M glycine and 0.3% Triton X-100, and nonspecific binding was blocked with 2% normal swine serum (DAKO, Glostrup, Denmark). Staining was performed as described previously^54^, using the primary anti-Phalloidin (Cytoskeleton Inc.) antibody. Samples were examined by fluorescence microscopy (Zeiss LSM 510 Meta). The calculation of expression was based on green fluorescence area and density divided by cell number, as determined from the number of DAPI-stained nuclei, in three randomly selected fields for each sample from a total of three independent experiments.

### Osteogenic differentiation

Endometrial stem cells were incubated in DMEM high-glucose medium supplemented with 0.1 µM dexamethasone, 10 mM β-glycerophosphate, 50 µM ascorbate and 10% FBS with or without GnRH. Endometrial stem cells were grown for 3 weeks, with medium replacement twice a week. Differentiated cells were stained with Alizarin Red S to detect de novo formation of bone matrix. Alizarin red S in samples was quantified by measuring the optical density (OD) of the solution at 570 nm.

### Real-time PCR

Total RNA from skin cells was extracted using TRIzol reagent (Invitrogen) according to the manufacturer’s protocol. The first-strand cDNA was synthesized with 1 to 2 μg of total RNA using SuperScript II (Invitrogen), and one-tenth of the cDNA was used for each PCR mixture containing Express SYBR-Green qPCR Supermix (BioPrince, Seoul, South Korea). Real-time PCR was performed using a Rotor-Gene Q (Qiagen). The reaction was subjected to amplification cycles of 95 °C for 20 s, 60 °C for 20 s and 72 °C for 25 s. The relative mRNA expression of the selected genes was normalized to that of PPIA and quantified using the ΔΔCT method. The sequences of the PCR primers are listed in supplementary table 1.

### Flow cytometry

FACS analysis and cell sorting were performed using FACS Calibur and FACS Aria machines (Becton Dickinson, Palo Alto, CA), respectively. FACS data were analyzed using FlowJo software (Tree Star, Ashland, OR). Antibodies to the following proteins were used: APC-conjugated CD44 (BD Bioscience, Cat. 559942, dilution 1/40), PE-conjugated CD133 (MACS; Miltenyi Biotech, Sunnyvale, CA, 130-080-081, dilution 1/40), CD34 (MACS; Miltenyi Biotech, Sunnyvale, CA, 30-081-002), CD44 (MACS; Miltenyi Biotech, Sunnyvale, CA, 130-095-180), CD45 (MACS; Miltenyi Biotech, Sunnyvale, CA, 130-080-201), CD73 (MACS; Miltenyi Biotech, Sunnyvale, CA, 130-095-182) and CD105 (MACS; Miltenyi Biotech, Sunnyvale, CA, 130-094-941) The FACS gates were established by staining with an isotype antibody or secondary antibody.

### Growth factor antibody array

The assay was performed following the manufacturer’s protocol (Abnova AA0089). Briefly, GnRH or vehicle-treated protein samples were incubated with antibody membranes overnight at 4 °C. After washing three times with wash buffer, the membranes were incubated with biotin-conjugated anti-cytokine antibodies overnight at 4 °C. The membranes were then washed 3 times and incubated with HRP-conjugated streptavidin. Chemiluminescence was used to detect signals of the growth factors spotted on the nitrocellulose membrane.

### GeneMANIA algorithm-based bioinformatics analysis

To further analyze genes that interact with or directly regulate PI3K/Akt signaling, we imported all identified genes and their corresponding accession numbers into GeneMANIA (http://www.genemania.org). To find gene interactions, we considered several factors including co-expression, co-localization, and genetic interactions. From this list, we selected the genes IGFBP-2, IGFBP-6, and IGF-I to test their involvement in regulating GRS-induced PI3K/Akt signaling.

### Evaluation of GnRH effects in animal model

All of the animal experiments were approved and carried out in accordance with the Institutional Animal Care and Use Committee (IACUC) (LCDI-2017-0102) of the Lee Gil Ya Cancer and Diabetes Institute of Gachon University. The mice were randomly divided into control (vehicle) and GnRH treatment groups. To exactly mimic clinical conditions, ICR mice were exposed to GnRH (0.1 mg/kg) or FSH (4 μg/kg) through intraperitoneal injection for 21 consecutive days similar to that in GnRH-based IVF protocol. The mice were anesthetized and exsanguinated by cardiac puncture, and then stem cells isolated from adipose tissues or uteri.

### Statistical analysis

All the statistical data were analyzed in GraphPad Prism 5.0 (GraphPad Software, San Diego, CA) and evaluated using two-tailed Student’s t-tests. Values of P < 0.05 were considered to indicate statistical significance.

## Electronic supplementary material

Supplementary figure 1

Supplementary figure 2

Supplementary figure 3

Supplementary figure 4

Supplementary figure 5

Supplementary figure 6

Supplementary figure 7

Supplementary figure 8

Supplementary information

## References

[1] Limonta P, Moretti RM, Marelli MM, Motta M (2003). The biology of gonadotropin hormone-releasing hormone: role in the control of tumor growth and progression in humans. Front. Neuroendocrinol..

[2] Neill JD (2002). GnRH and GnRH receptor genes in the human genome. Endocrinology.

[3] Kumar P, Sharma A (2014). Gonadotropin-releasing hormone analogs: Understanding advantages and limitations. J. Hum. Reprod. Sci..

[4] Depalo R (2012). GnRH agonist versus GnRH antagonist in in vitro fertilization and embryo transfer (IVF/ET). Reprod. Biol. Endocrinol..

[5] Lai Q (2013). Comparison of the GnRH agonist and antagonist protocol on the same patients in assisted reproduction during controlled ovarian stimulation cycles. Int. J. Clin. Exp. Pathol..

[6] Lucas ES, Salker MS, Brosens JJ (2013). Uterine plasticity and reproductive fitness. Reprod. Biomed..

[7] McLennan CE, Rydell AH (1965). Extent of endometrial shedding during normal menstruation. Obstet. Gynecol..

[8] Omidvar S, Begum K (2011). Menstrual pattern among unmarried women from south India. J. Nat. Sci. Biol. Med..

[9] Lai TH, Chang FW, Lin JJ, Ling QD (2018). Gene expression of human endometrial L-selectin ligand in relation to the phases of the natural menstrual cycle. Sci. Rep..

[10] Sanderson PA, Critchley HO, Williams AR, Arends MJ, Saunders PT (2017). New concepts for an old problem: the diagnosis of endometrial hyperplasia. Hum. Reprod. Update.

[11] Gargett CE, Nguyen HP, Ye L (2012). Endometrial regeneration and endometrial stem/progenitor cells. Rev. Endocr. Metab. Disord..

[12] Gargett CE, Ye L (2012). Endometrial reconstruction from stem cells. Fertil. Steril..

[13] Lucas ES (2016). Loss of Endometrial Plasticity in Recurrent Pregnancy Loss. Stem Cells.

[14] Khodr GS, Siler-Khodr TM (1980). Placental luteinizing hormone-releasing factor and its synthesis. Science.

[15] Kang SK, Tai CJ, Nathwani PS, Leung PC (2001). Differential regulation of two forms of gonadotropin-releasing hormone messenger ribonucleic acid in human granulosa-luteal cells. Endocrinology.

[16] Irmer G, Burger C, Ortmann O, Schulz KD, Emons G (1994). Expression of luteinizing hormone releasing hormone and its mRNA in human endometrial cancer cell lines. J. Clin. Endocrinol. Metab..

[17] Weng H, Liu F, Hu S, Li L, Wang Y (2014). GnRH agonists induce endometrial epithelial cell apoptosis via GRP78 downregulation. J. Transl. Med..

[18] Ersoy GS, Zolbin MM, Cosar E, Mamillapalli R, Taylor HS (2017). Medical Therapies for Endometriosis Differentially Inhibit Stem Cell Recruitment. Reprod. Sci..

[19] Forte G (2006). Hepatocyte growth factor effects on mesenchymal stem cells: proliferation, migration, and differentiation. Stem Cells.

[20] Gharibi B, Ghuman MS, Hughes FJ (2012). Akt- and Erk-mediated regulation of proliferation and differentiation during PDGFRbeta-induced MSC self-renewal. J. Cell Mol. Med..

[21] Zheng B (2013). Neural differentiation of mesenchymal stem cells influences chemotactic responses to HGF. J. Cell Physiol..

[22] Song BQ (2015). Inhibition of Notch signaling promotes the adipogenic differentiation of mesenchymal stem cells through autophagy activation and PTEN-PI3K/AKT/mTOR pathway. Cell Physiol. Biochem..

[23] Tang JM (2012). Acetylcholine induces mesenchymal stem cell migration via Ca2+/PKC/ERK1/2 signal pathway. J. Cell Biochem..

[24] Yamaguchi H, Condeelis J (2007). Regulation of the actin cytoskeleton in cancer cell migration and invasion. Biochim. Biophys. Acta.

[25] Agrawal R, Holmes J, Jacobs HS (2000). Follicle-stimulating hormone or human menopausal gonadotropin for ovarian stimulation in in vitro fertilization cycles: a meta-analysis. Fertil. Steril..

[26] Conn PM, Crowley WF (1994). Gonadotropin-releasing hormone and its analogs. Annu. Rev. Med..

[27] Sealfon SC, Weinstein H, Millar RP (1997). Molecular mechanisms of ligand interaction with the gonadotropin-releasing hormone receptor. Endocr. Rev..

[28] Armstrong L (2006). The role of PI3K/AKT, MAPK/ERK and NFkappabeta signalling in the maintenance of human embryonic stem cell pluripotency and viability highlighted by transcriptional profiling and functional analysis. Hum. Mol. Genet..

[29] Muller P, Langenbach A, Kaminski A, Rychly J (2013). Modulating the actin cytoskeleton affects mechanically induced signal transduction and differentiation in mesenchymal stem cells. PLoS ONE.

[30] Gao F, Hu X, Xie X, Liu X, Wang J (2015). Heat shock protein 90 stimulates rat mesenchymal stem cell migration via PI3K/Akt and ERK1/2 pathways. Cell Biochem. Biophys..

[31] Myers AL (2015). IGFBP2 modulates the chemoresistant phenotype in esophageal adenocarcinoma. Oncotarget.

[32] Yang Y (2014). Downregulation of Insulin-like growth factor binding protein 6 is associated with ACTH-secreting pituitary adenoma growth. Pituitary.

[33] Aplin JD, Ruane PT (2017). Embryo-epithelium interactions during implantation at a glance. J. Cell Sci..

[34] Altmae S (2012). Research resource: interactome of human embryo implantation: identification of gene expression pathways, regulation, and integrated regulatory networks. Mol. Endocrinol..

[35] Demir R, Yaba A, Huppertz B (2010). Vasculogenesis and angiogenesis in the endometrium during menstrual cycle and implantation. Acta Histochem..

[36] Morelli SS, Yi P, Goldsmith LT (2012). Endometrial stem cells and reproduction. Obstet. Gynecol. Int..

[37] Lucas ES, Dyer NP, Fishwick K, Ott S, Brosens JJ (2016). Success after failure: the role of endometrial stem cells in recurrent miscarriage. Reproduction.

[38] Maggi R., et al. GnRH and GnRH receptors in the pathophysiology of the human female reproductive system. *Hum Reprod Update* **22**, 358–381 (2016). 10.1093/humupd/dmv059 26715597

[39] Metallinou C, Asimakopoulos B, Schroer A, Nikolettos N (2007). Gonadotropin-releasing hormone in the ovary. Reprod. Sci..

[40] Casan EM, Raga F, Bonilla-Musoles F, Polan ML (2000). Human oviductal gonadotropin-releasing hormone: possible implications in fertilization, early embryonic development, and implantation. J. Clin. Endocrinol. Metab..

[41] Wolfahrt S, Kleine B, Jarry H, Rossmanith WG (2001). Endogenous regulation of the GnRH receptor by GnRH in the human placenta. Mol. Hum. Reprod..

[42] Tieva A, Stattin P, Wikstrom P, Bergh A, Damber JE (2001). Gonadotropin-releasing hormone receptor expression in the human prostate. Prostate.

[43] Clayton RN, Katikineni M, Chan V, Dufau ML, Catt KJ (1980). Direct inhibition of testicular function by gonadotropin-releasing hormone: mediation by specific gonadotropin-releasing hormone receptors in interstitial cells. Proc. Natl Acad. Sci. USA.

[44] Chen A (2002). Two forms of gonadotropin-releasing hormone (GnRH) are expressed in human breast tissue and overexpressed in breast cancer: a putative mechanism for the antiproliferative effect of GnRH by down-regulation of acidic ribosomal phosphoproteins P1 and P2. Cancer Res..

[45] Wu HM (2009). Gonadotropin-releasing hormone type II induces apoptosis of human endometrial cancer cells by activating GADD45alpha. Cancer Res..

[46] Tsui KH (2014). Effect of gonadotropin-releasing hormone agonist on ES-2 ovarian cancer cells. Taiwan. J. Obstet. Gynecol..

[47] Kraus S, Naor Z, Seger R (2006). Gonadotropin-releasing hormone in apoptosis of prostate cancer cells. Cancer Lett..

[48] Meresman GF (2003). Gonadotropin-releasing hormone agonist induces apoptosis and reduces cell proliferation in eutopic endometrial cultures from women with endometriosis. Fertil. Steril..

[49] Borroni R (2000). Expression of GnRH receptor gene in human ectopic endometrial cells and inhibition of their proliferation by leuprolide acetate. Mol. Cell Endocrinol..

[50] van der Linden M., Buckingham K., Farquhar C., Kremer J. A., Metwally M. Luteal phase support for assisted reproduction cycles. *Cochrane Database Syst. Rev*. CD009154 (2015). 10.1002/14651858.CD009154.pub3 PMC6461197 26148507

[51] Hossini AM (2016). PI3K/AKT signaling pathway is essential for survival of induced pluripotent stem cells. PLoS ONE.

[52] Chen J, Crawford R, Chen C, Xiao Y (2013). The key regulatory roles of the PI3K/Akt signaling pathway in the functionalities of mesenchymal stem cells and applications in tissue regeneration. Tissue Eng. Part B Rev..

[53] Choi ES (2013). Myeloid cell leukemia-1 is a key molecular target for mithramycin A-induced apoptosis in androgen-independent prostate cancer cells and a tumor xenograft animal model. Cancer Lett..

[54] Dong HJ (2016). The Wnt/beta-catenin signaling/Id2 cascade mediates the effects of hypoxia on the hierarchy of colorectal-cancer stem cells. Sci. Rep..

