# Supplementary figures and images for: Double-edged sword of gonadotropin-releasing hormone (GnRH): A novel role of GnRH in the multiple beneficial functions of endometrial stem cells
Source: Cell Death Dis. 2018 Aug 1;9(8):828. doi: 10.1038/s41419-018-0892-3 (PMC6070560; doi:10.1038/s41419-018-0892-3)

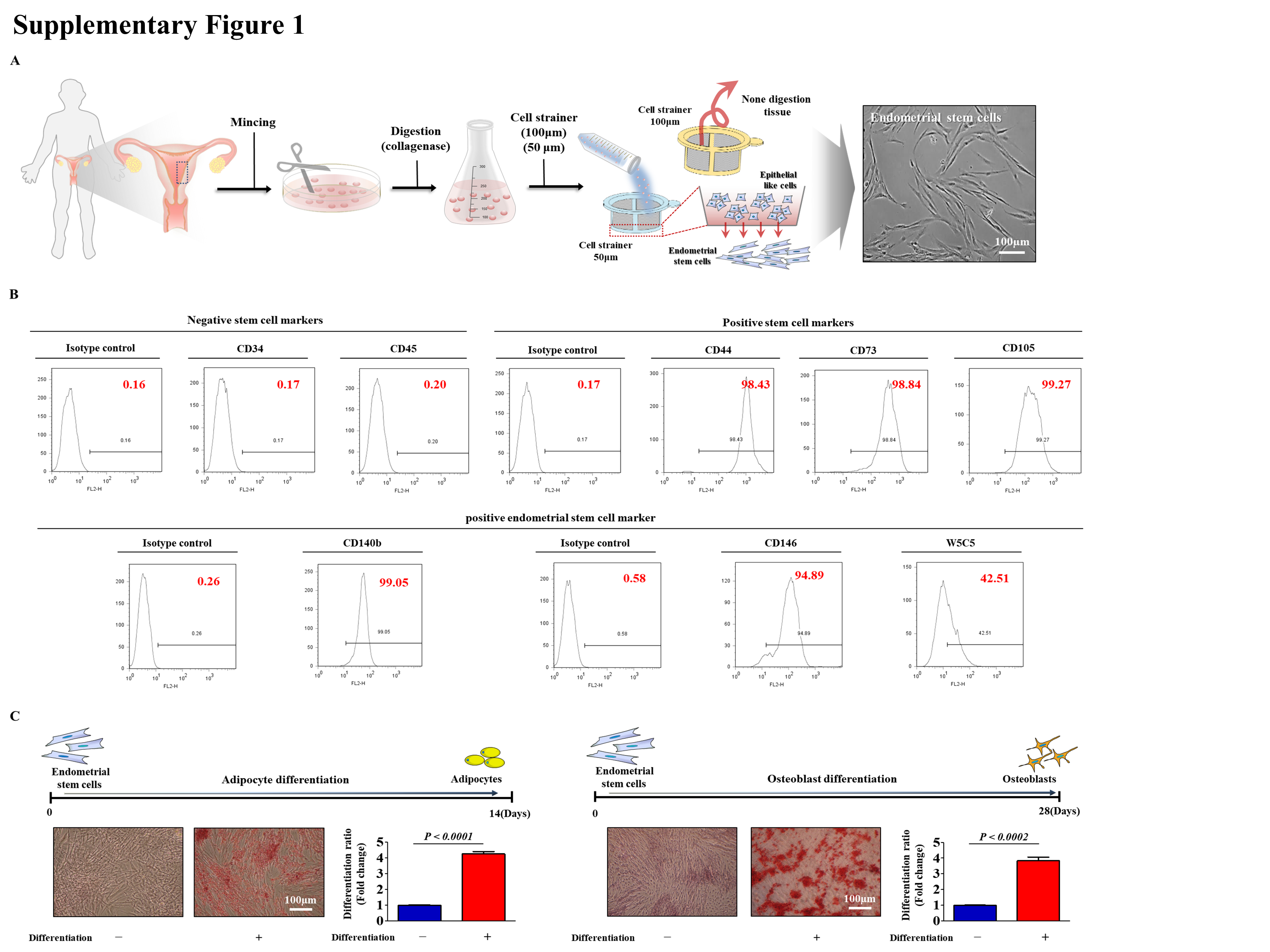

Supplement: Supplementary file 1 — Supplementary figure 1 [file 41419_2018_892_MOESM1_ESM.tif]

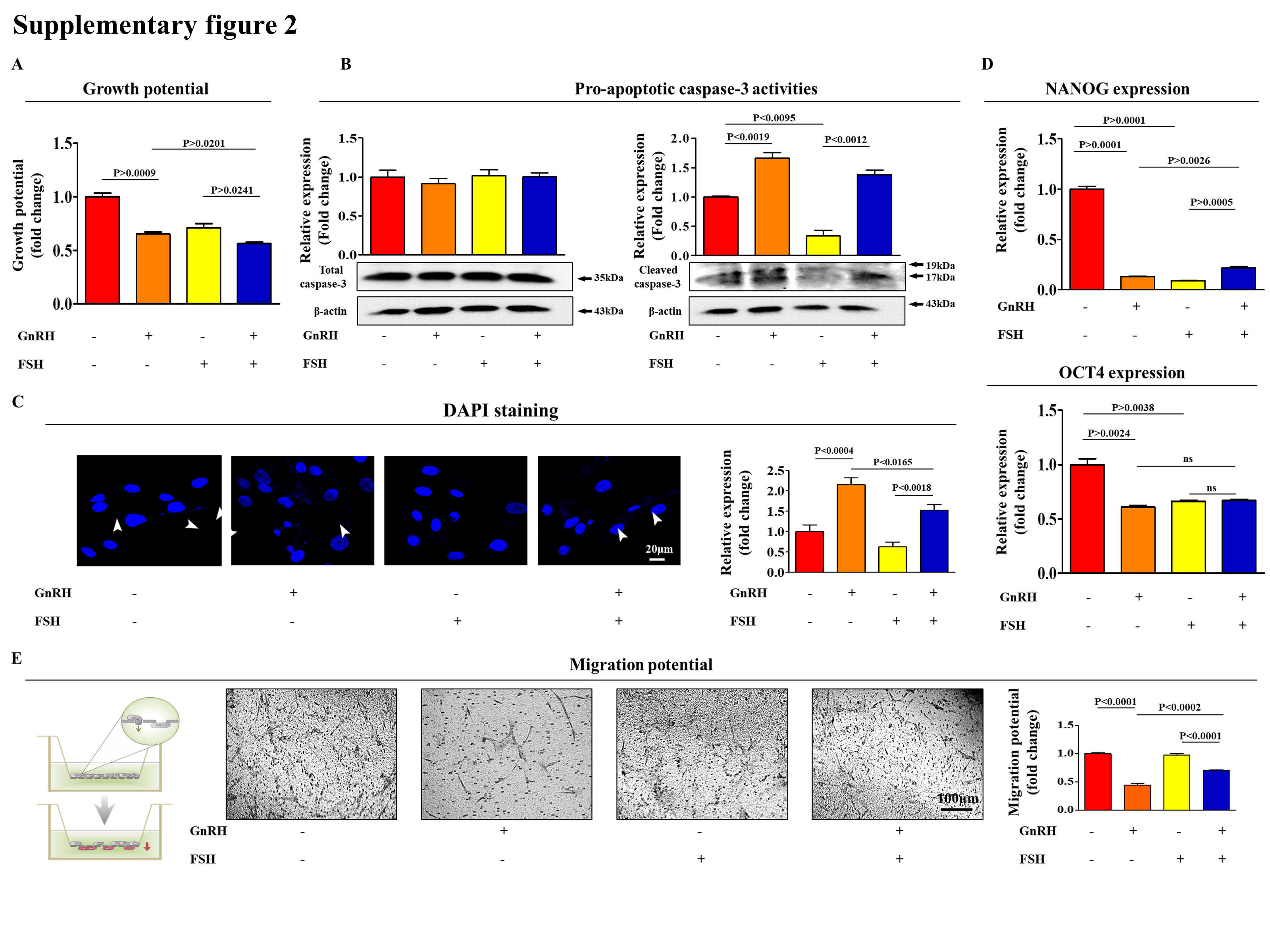

Supplement: Supplementary file 2 — Supplementary figure 2 [file 41419_2018_892_MOESM2_ESM.tif]

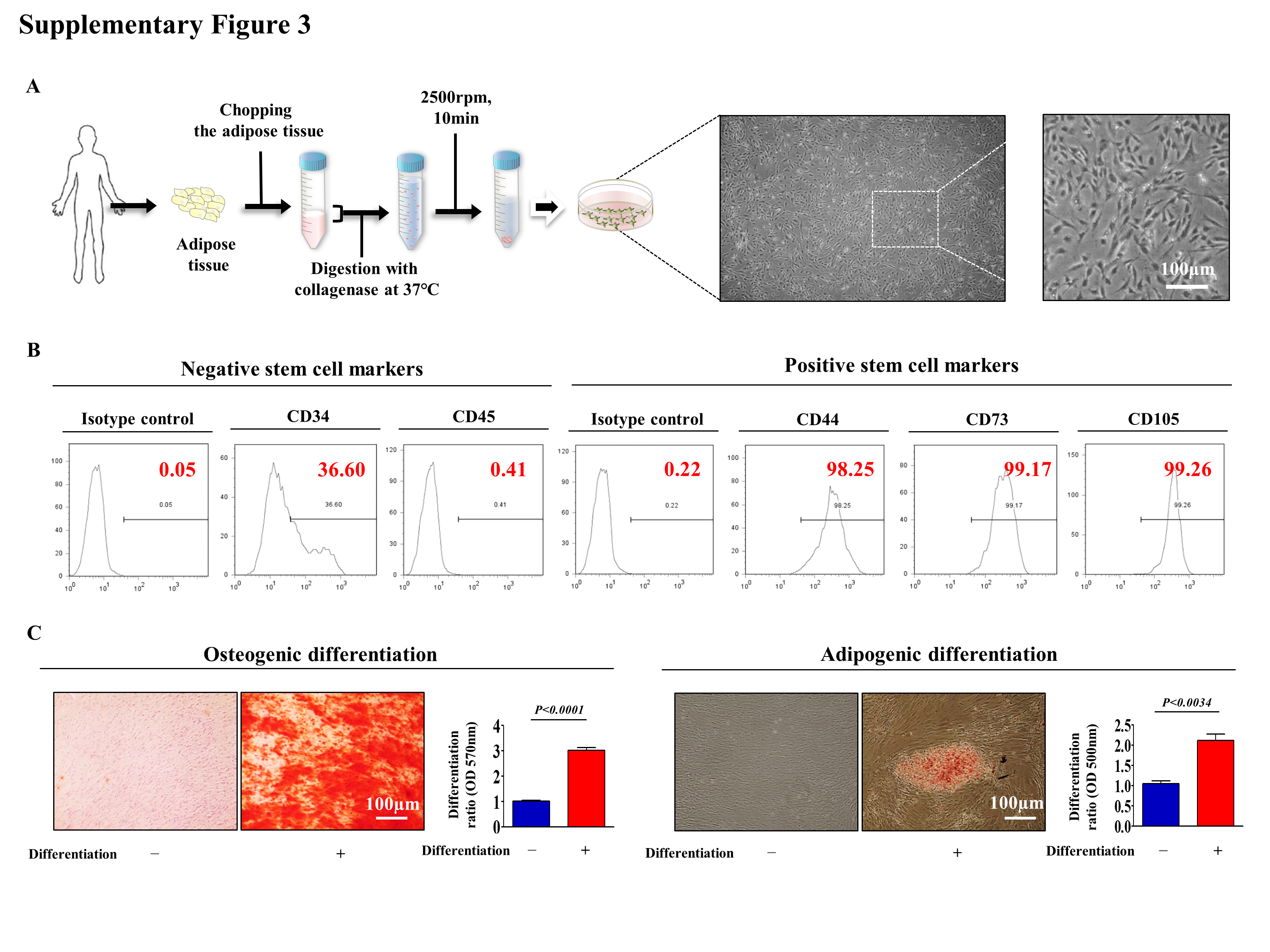

Supplement: Supplementary file 3 — Supplementary figure 3 [file 41419_2018_892_MOESM3_ESM.tif]

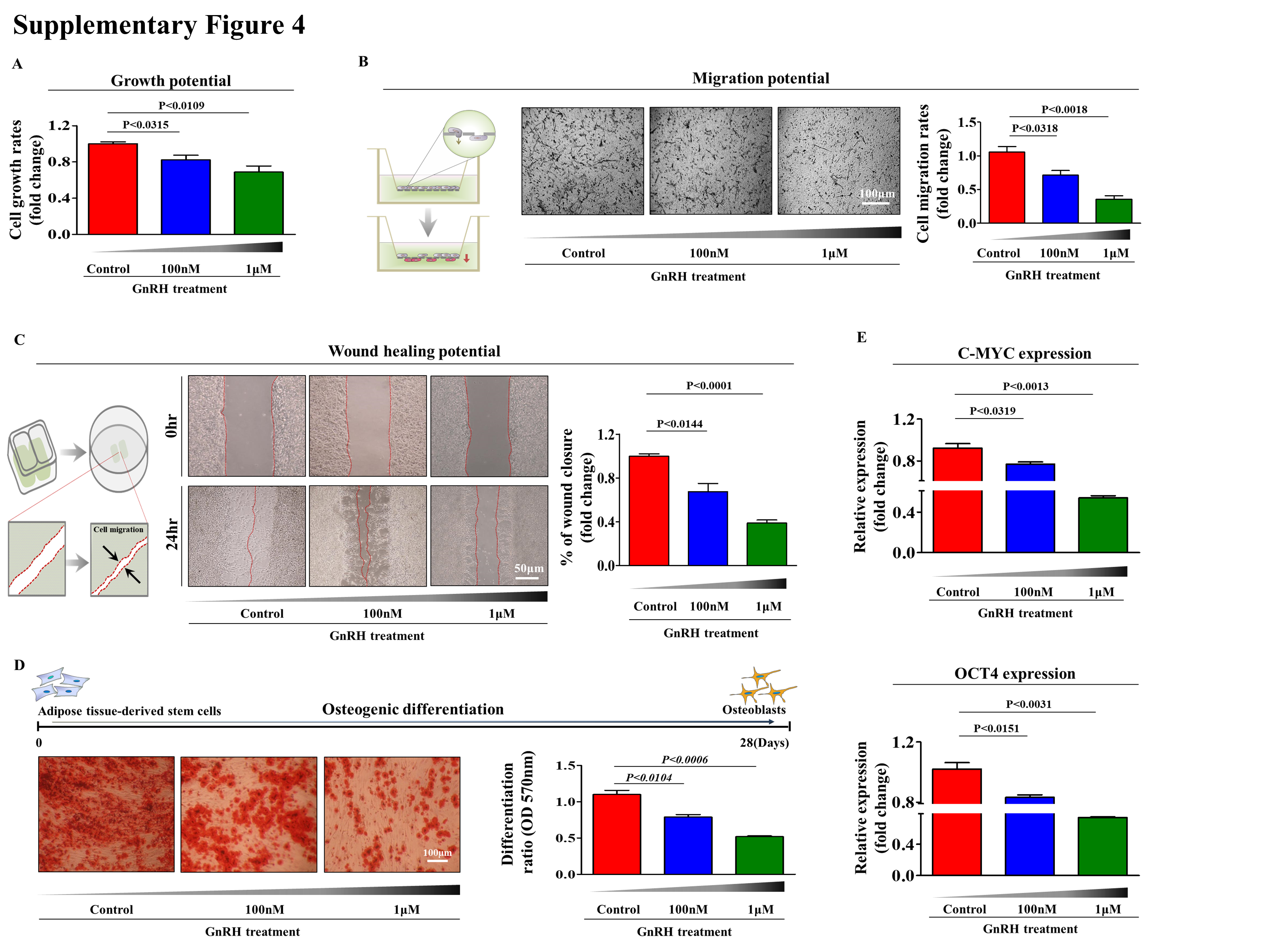

Supplement: Supplementary file 4 — Supplementary figure 4 [file 41419_2018_892_MOESM4_ESM.tif]

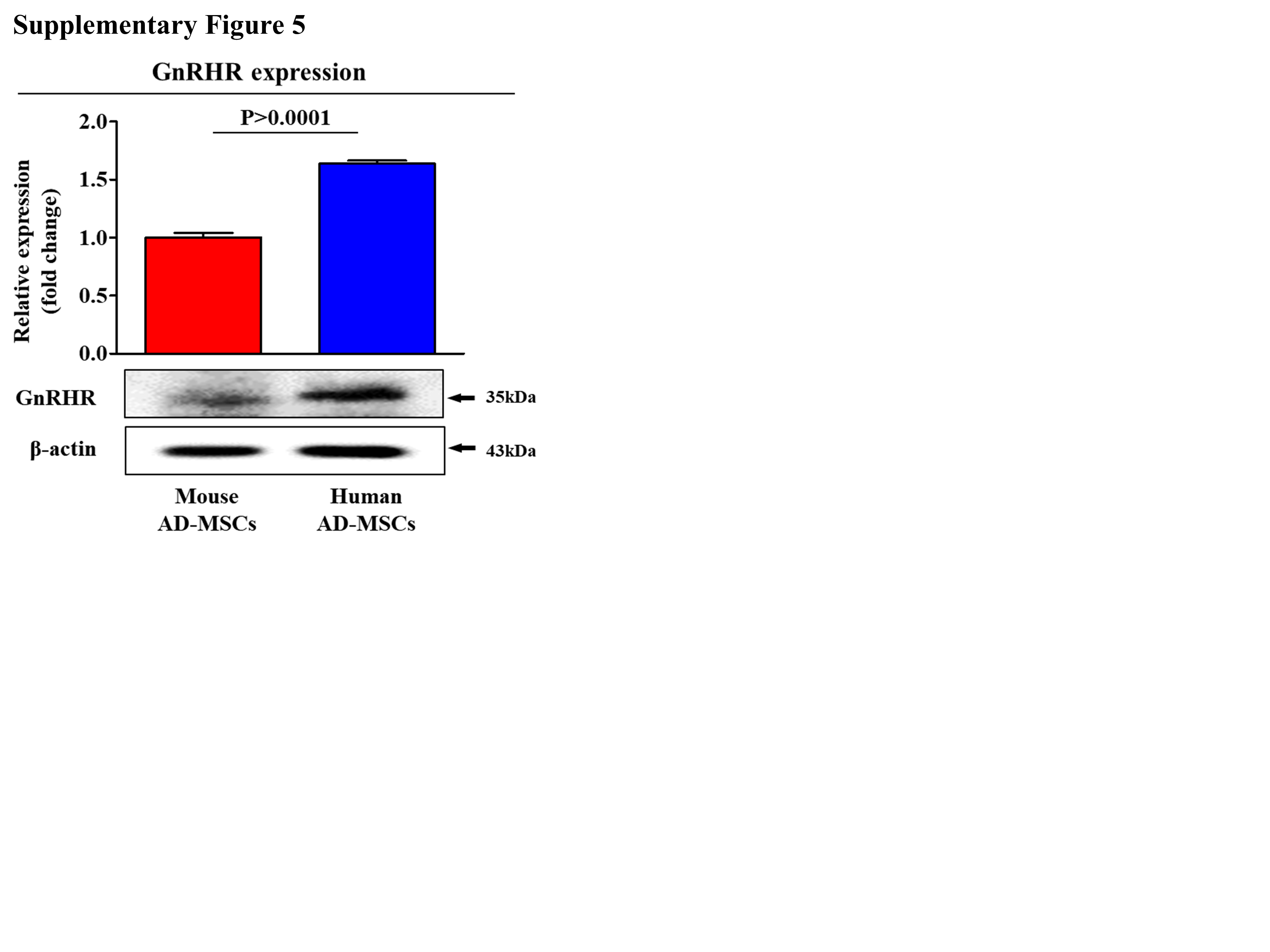

Supplement: Supplementary file 5 — Supplementary figure 5 [file 41419_2018_892_MOESM5_ESM.tif]

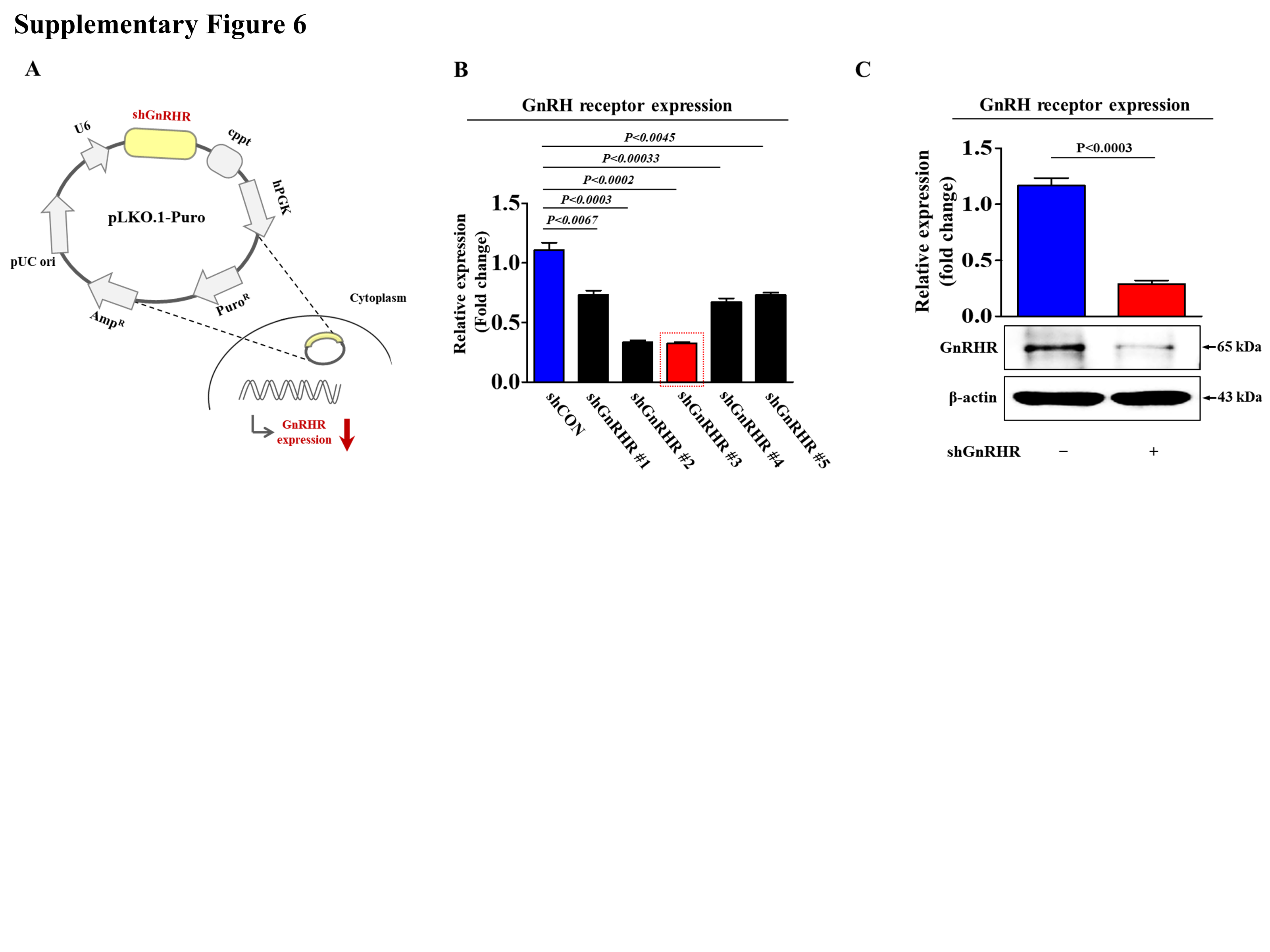

Supplement: Supplementary file 6 — Supplementary figure 6 [file 41419_2018_892_MOESM6_ESM.tif]

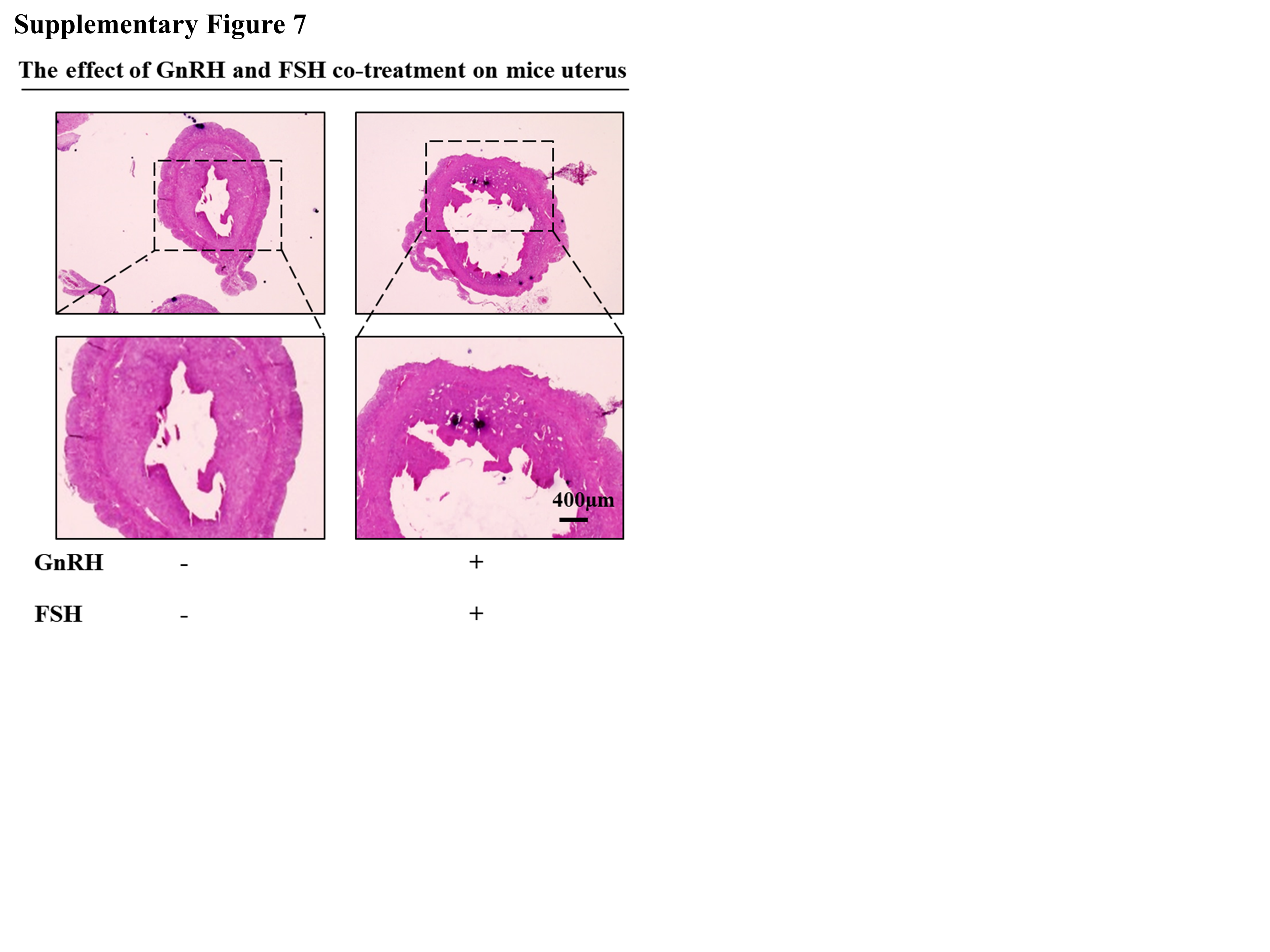

Supplement: Supplementary file 7 — Supplementary figure 7 [file 41419_2018_892_MOESM7_ESM.tif]

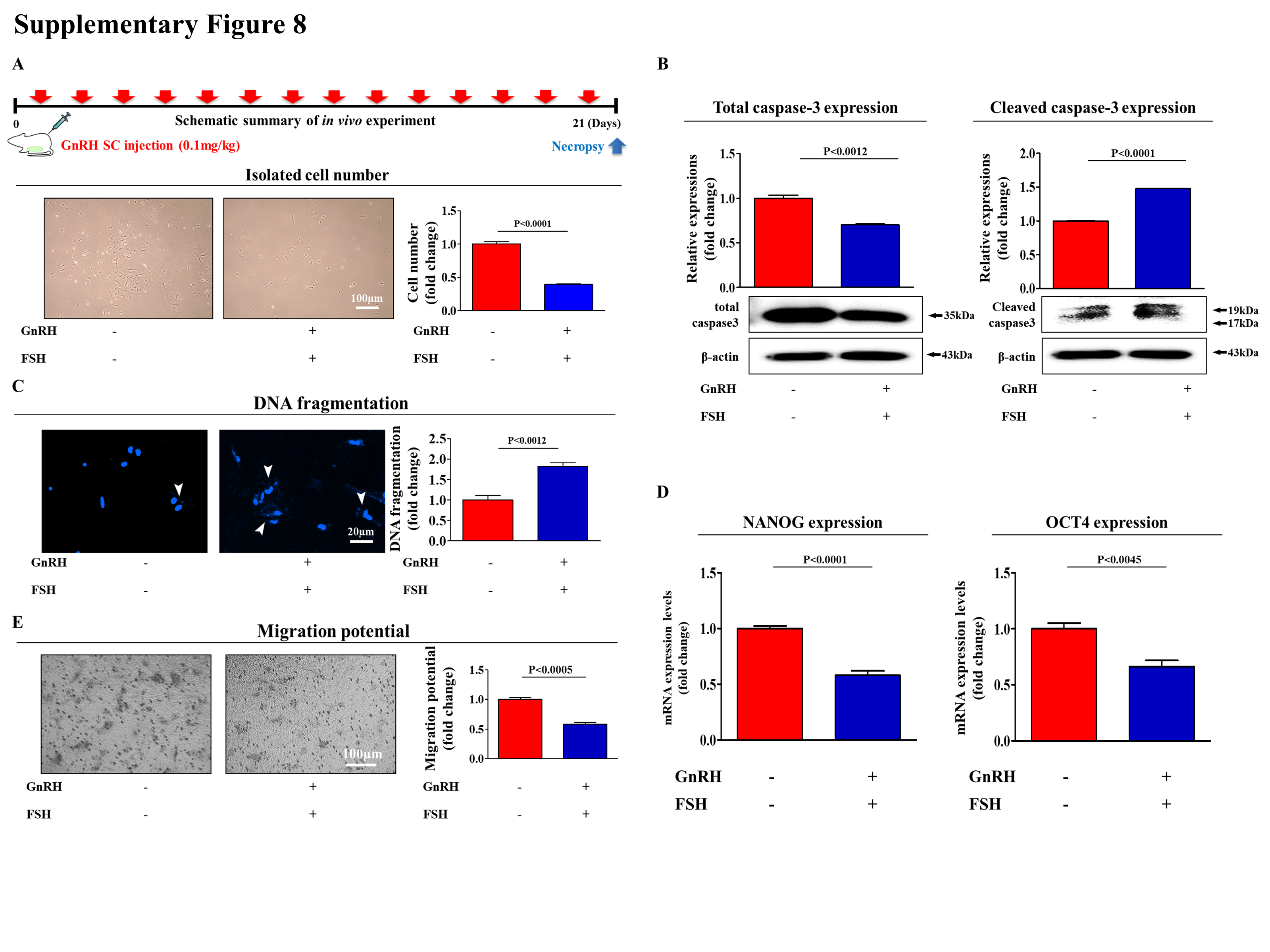

Supplement: Supplementary file 8 — Supplementary figure 8 [file 41419_2018_892_MOESM8_ESM.tif]
