# Supplementary material for: Double-edged sword of gonadotropin-releasing hormone (GnRH): A novel role of GnRH in the multiple beneficial functions of endometrial stem cells
Source: Cell Death Dis. 2018 Aug 1;9(8):828. doi: 10.1038/s41419-018-0892-3 (PMC6070560; doi:10.1038/s41419-018-0892-3)
Supplement: Supplementary file 9 — Supplementary information [file 41419_2018_892_MOESM9_ESM.docx]

**Supplementary figure legends**

**Supplementary figure 1. Isolation and characterization of human endometrial stem cells.** Spindle-shaped human endometrial stem cells can be observed by phase-contrast microscopy **(A)**. The isolated cells were positive for the stem cell markers CD44, CD73, CD105, CD140b, CD146, and W5C5 as well as negative for the hematopoietic markers CD34 and CD45 (B). The capacity of these stem cells to differentiate into multiple lineages, specifically osteoblasts and adipocytes, was determined by alizarin red staining and oil red O staining, respectively. The relative quantification of calcium mineral content and lipid droplet formation was performed by measuring the absorbance at 570 nm and 500 nm, respectively **(C)**. The data are presented as the mean ± SD of three independent experiments.

**Supplementary figure 2.** **The effects of GnRH and FSH co-treatment on multiple beneficial functions of endometrial stem cells *in vitro*.** The inhibition of endometrial stem cell viability by GnRH (1 µM) and FSH (50ng/ml) co-treatment at 72 h was determined by an MTT assay. Stem cell viability (%) was calculated as a percent of the vehicle control **(A)**. Elevated levels of cleaved caspase-3 following GnRH and FSH co-treatment were assessed by western blotting **(B)**. GnRH and FSH cotreatment-induced apoptotic DNA fragmentation and condensation were visualized using DAPI staining **(C)**. Real-time PCR results showed the changes in the expression of the stem cell markers NANOG, OCT4, and SOX2 after GnRH treatment for 72 h **(D)**. Endometrial stem cells were treated with GnRH and FSH for 72 h, and the effect of co-treatment on stem cell migration ability was then evaluated using the transwell migration assay **(E)**. β-actin was used as the internal control. The data are presented as the mean ± SD of three independent experiments.

**Supplementary figure 3. Isolation and characterization of human adipose tissue-derived stem cells.** Spindle-shaped human adipose tissue-derived stem cells can be observed by phase-contrast microscopy **(A)**. The isolated cells were positive for the stem cell markers CD44, CD73, and CD105 and negative for the hematopoietic markers CD34 and CD45 **(B)**. The capacity of these stem cells to differentiate into multiple lineages, specifically osteoblasts and adipocytes, was determined by alizarin red staining and oil red O staining, respectively. The relative quantification of calcium mineral content and lipid droplet formation was performed by measuring the absorbance at 570 nm and 500 nm, respectively **(C)**. The data are presented as the mean ± SD of three independent experiments.

**Supplementary figure 4. GnRH suppresses multiple beneficial functions of adipose tissue-derived stem cells *in vitro*.** The inhibition of stem cell viability by 1 µM GnRH at 72 h was determined by an MTT assay. Stem cell viability (%) was calculated as a percent of the vehicle control **(A)**. Stem cells were treated with GnRH for 72 h, and the effect of GnRH on cell migration was then evaluated using the transwell migration assay. GnRH treatment significantly decreased stem cell migration across the membrane compared with the negative control **(B)**. The effects of GnRH on stem cell migration were further evaluated using a scratch assay. The migration of GnRH-treated stem cells was slower than that of vehicle-treated cells **(C)**. Confluent stem cells were cultured in osteogenic medium with or without GnRH. The effects of GnRH on osteoblast differentiation were determined by alizarin red staining. The relative quantification of calcium mineral content was performed by measuring the absorbance at 570 nm **(D)**. Real-time PCR results showed the changes in the expression of the stem cell markers C-MYC and OCT4 after GnRH treatment for 72 h **(E)**. The data are presented as the mean ± SD of three independent experiments.

**Supplementary figure 5. GnRH receptor expression profiles in both human and mouse adipose derived stem cells.** GnRH receptor expression was confirmed by western blotting in both human and mouse adipose derived stem cells. Both stem cells expressed GnRH receptors although the expression was more marked in human stem cells than mouse stem cells **(A)**. β-actin was used as the internal control. The data are presented as the mean ± SD of three independent experiments.

**Supplementary figure 6. Knockdown efficacy of shRNAs targeting GnRH-R.** Endometrial stem cells were stably transduced with shRNA #1, #2, #3, #5, or #4, which target GnRH-R, or with a non-targeting control shRNA **(A)**. GnRH-R shRNA construct #3, hereafter referred to as GnRH-R shRNA, was the most effective. The successful knockdown of GnRH-R expression was verified based on RNA **(B)** and protein expression levels **(C)** in endometrial stem cells. β-actin was used as the internal control. The data are presented as the mean ± SD of three independent experiments.

**Supplementary figure 7. GnRH treatment produced degenerative changes of endometrium *in vivo*.** Mice were treated daily for 21 days with GnRH (0.1 mg/kg, intraperitoneally) or vehicle (PBS). Uteri were removed and then hematoxylin and eosin (H&E) staining was performed, and the tissues were analyzed for the histopathological examination.

**Supplementary figure 8.** **The effects of GnRH and FSH co-treatment on multiple beneficial functions of endometrial stem cells *in vivo*.** Schematic representation of the experimental protocol as described in the materials and methods section **(A)**. Mice were treated daily for 21 days with GnRH (0.1 mg/kg, intraperitoneally) or FSH (4μg/kg, intraperitoneally). Stem cells were isolated from mouse uterus and then the inhibition of endometrial stem cell viability was determined by an MTT assay. Stem cell viability (%) was calculated as a percent of the vehicle control **(A)**. Elevated levels of cleaved caspase-3 following GnRH and FSH co-treatment were assessed by western blotting **(B)**. GnRH and FSH co-treatment-induced apoptotic DNA fragmentation and condensation were visualized using DAPI staining **(C)**. Real-time PCR results showed the changes in the expression of the stem cell markers NANOG, OCT4, and SOX2 after GnRH treatment for 72 h **(D)**. Endometrial stem cells were treated with GnRH and FSH for 72 h, and the effect of co-treatment on stem cell migration ability was then evaluated using the transwell migration assay **(E)**. β-actin was used as the internal control. The data are presented as the mean ± SD of three independent experiments.

**Supplementary table 1. Primer sequences for quantitative RT-PCR.**

| **Gene** | **Gene bank No.** | **Direction** | **Primer sequence** |
| --- | --- | --- | --- |
| Human PPIA NM_021130 | | F | TGCCATCGCCAAGGAGTAG |
|  |  | R | TGCACAGACGGTCACTCAAA |
| Human C-MYC | NM_002467 | F | AAAGGCCCCCAAGGTAGTTA |
|  |  | R | GCACAAGAGTTCCGTAGCTG |
| Human NANOG | NM_024865 | F | ACATGCAACCTGAAGACGTGTG |
|  |  | R | CATGGAAACCAGAACACGTGG |
| Human OCT4 | NM_002701 | F | AGCCCTCATTTCACCAGGCC |
|  |  | R | TGGGACTCCTCCGGGTTTTG |
| Human GnRH receptor | NM_000406 | F | CCATGTATGCCCCAGCCTT |
|  |  | R | GCCTCGTGATAGCCAGGGA |
| Mouse HPRT NM_013556 | | F | GCCTAAGATGAGCGCAAGTTG |
|  |  | R | TACTAGGCAGATGGCCACAGG |
| Mouse C-MYC | NM_010849 | F | CGCACACACAACGTCTTGGA |
|  |  | R | AGGATGTAGGCGGTGGCTTT |
| Mouse KLF4 NM_010637 | | F | GGTGCAGCTTGCAGCAGTAA |
|  |  | R | AAAGTCTAGGTCCAGGAGGT |
